# Staggered Hox expression is more widespread among molluscs than previously appreciated

**DOI:** 10.1098/rspb.2018.1513

**Published:** 2018-10-10

**Authors:** Tim Wollesen, Sonia Victoria Rodríguez Monje, André Luiz de Oliveira, Andreas Wanninger

**Affiliations:** Department of Integrative Zoology, Faculty of Life Sciences, University of Vienna, Althanstraße 14, 1090 Vienna, Austria

**Keywords:** cephalopod, gastropod, gene expression, Lophotrochozoa, nervous system, Polyplacophora

## Abstract

Hox genes are expressed along the anterior–posterior body axis in a colinear fashion in the majority of bilaterians. Contrary to polyplacophorans, a group of aculiferan molluscs with conserved ancestral molluscan features, gastropods and cephalopods deviate from this pattern by expressing Hox genes in distinct morphological structures and not in a staggered fashion. Among conchiferans, scaphopods exhibit many similarities with gastropods, cephalopods and bivalves, however, the molecular developmental underpinnings of these similar traits remain unknown. We investigated Hox gene expression in developmental stages of the scaphopod *Antalis entalis* to elucidate whether these genes are involved in patterning morphological traits shared by their kin conchiferans. Scaphopod Hox genes are predominantly expressed in the foot and mantle but also in the central nervous system. Surprisingly, the scaphopod mid-stage trochophore exhibits a near-to staggered expression of all nine Hox genes identified. Temporal colinearity was not found and early-stage and late-stage trochophores, as well as postmetamorphic individuals, do not show any apparent traces of staggered expression. In these stages, Hox genes are expressed in distinct morphological structures such as the cerebral and pedal ganglia and in the shell field of early-stage trochophores. Interestingly, a re-evaluation of previously published data on early-stage cephalopod embryos and of the gastropod pre-torsional veliger shows that these developmental stages exhibit traces of staggered Hox expression. Considering our results and all gene expression and genomic data available for molluscs as well as other bilaterians, we suggest a last common molluscan ancestor with colinear Hox expression in predominantly ectodermal tissues along the anterior–posterior axis. Subsequently, certain Hox genes have been co-opted into the patterning process of distinct structures (apical organ or prototroch) in conchiferans.

## 1. Introduction

**Figure 1.**
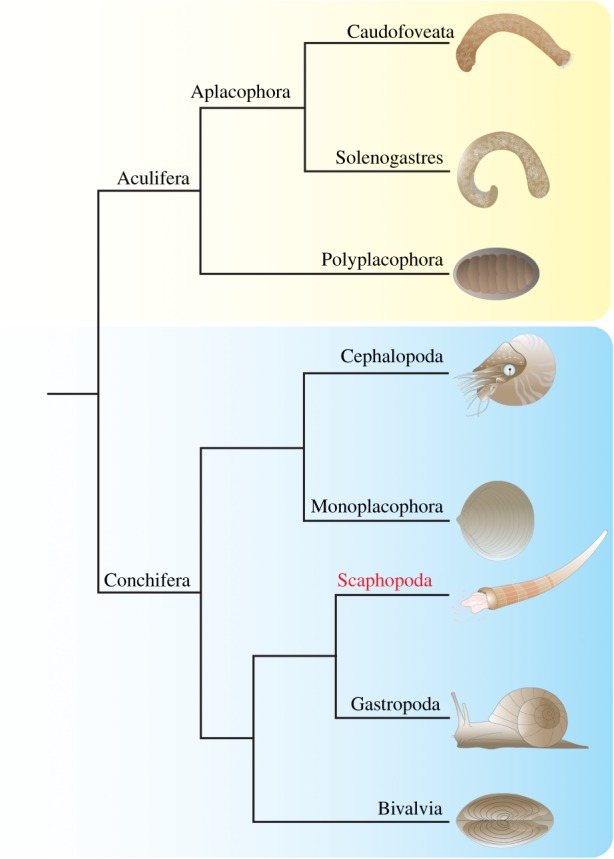
Current view of molluscan phylogeny based on Smith *et al.* [3]. Aculifera and Conchifera are sister groups. Aculiferans comprise the eight-shelled Polyplacophora with its sister group composed of the worm-shaped Solenogastres and Caudofoveata. Conchiferans are an assemblage of cephalopods/monoplacophorans and a sister group comprising bivalves as well as a scaphopod/gastropod clade.

There are few organismal groups that exhibit a comparably diverse fossil record and extant phenotypic diversity as the Mollusca. Ancestors of this ancient lineage probably already populated Ediacaran oceans around 550 Ma [1,2]. Modern molluscan clades such as the conchiferan gastropods, scaphopods and cephalopods or the aculiferan polyplacophorans therefore have evolved over a long time into their very diverse recent body shapes (figure 1; [4]). Scaphopoda is a rather small molluscan taxon that is characterized by a tusk-shaped shell, a pronounced foot and a cephalic region equipped with numerous tentacles, called captacula (figure 1, [5]). Although little investigated, it constitutes a key taxon to infer the evolution of conchiferan body plans because it exhibits ontogenetic and morphological features resembling the gastropod, bivalve and cephalopod condition. Scaphopods are eyeless but possess a buccal cone with a radula and hence show an intermediate condition between the ‘headless’ bivalves, on the one hand, and the gastropods and cephalopods with distinct heads, on the other [6]. Bivalves and scaphopods exhibit a mantle that engulfs the pronounced foot and gives rise to the shell field(s) of early developmental stages. Adult scaphopods and cephalopods possess a prominent dorsal–ventral body axis and a u-shaped gut (the latter also being present in gastropods). The attachment sites of the dorsal–ventral muscles are ring-shaped, and cephalopods and scaphopods possess numerous tentacles, i.e. the cephalopod arms and the scaphopod captacula. In addition, there are also similarities on the molecular level [6] and recent studies showed that in both taxa the ParaHox gene *Gsx* is only expressed in the central nervous system and not in association with the oesophagus as shown for gastropods and some other bilaterians [7,8]. All conchiferans except cephalopods also possess a similar topological arrangement of cerebral, pedal, pleural and buccal ganglia (the latter have been lost in Bivalvia) and a trochophore-like larva is present in all molluscs except for the cephalopods that exhibit direct development (the life cycle of monoplacophorans remains unknown).

Individual morphological structures such as ganglia, the larval prototroch and the apical organ, but also lineage-specific traits such as the cephalopod funnel and arms, have been shown to express Hox genes [7,9–11]. By encoding transcription factors which regulate downstream target genes, Hox genes pattern the body plan during ontogeny [12,13]. Developmental changes of Hox gene regulation may lead to significant morphological changes which may result in the evolution of new taxa [14,15]. While no staggered Hox expression has been reported for gastropods and cephalopods so far, recent studies showed this condition for the aculiferan polyplacophoran *Acanthochitona crinita* [16,17]. Staggered expression implies that during ontogeny Hox genes are expressed in a subsequent spatial order along the anterior–posterior body axis. If this expression matches the genes' relative position on the chromosome, then it is referred to as spatial colinearity. The latter is often accompanied by temporal colinearity, i.e. anterior Hox genes are expressed earlier during ontogeny than more posterior ones [18]. Since representatives of all three bilaterian superphyla (Lophotrochozoa, Ecdysozoa, Deuterostomia) exhibit colinear Hox expression, it may be assumed that this condition was already present in the last common ancestor of Bilateria.

In order to elucidate whether Hox and ParaHox genes are expressed in a staggered fashion and/or in homologous morphological traits of molluscs, we performed gene expression analyses during the ontogeny of the scaphopod *Antalis entails*.

## 2. Material and methods

Animals were collected, cultured and fixed as described previously (see the electronic supplementary material, material and methods section; [8]). RNA was extracted and sequenced, the phylogenetic analysis, cloning, probe synthesis, *in situ* hybridization and the documentation of samples were described in detail previously (see the electronic supplementary material, material and methods section).

## 3. Results

### (a) Hox and ParaHox gene expression in the scaphopod *Antalis entalis*

Hox and ParaHox genes have been previously identified based on a transcriptome screen of pooled developmental stages of the scaphopod *An. entalis* [19]. Thereby, all predicted Hox and ParaHox genes except for *Hox7*/*Antp* and *Xlox* were found. Unfortunately, we were unable to clone *Lox2*. Scaphopod Hox genes are expressed from the gastrula stage (not shown) to early postmetamorphic individuals (figures 2–5; electronic supplementary material, figures S3–20; adults were not investigated).

**Figure 2.**
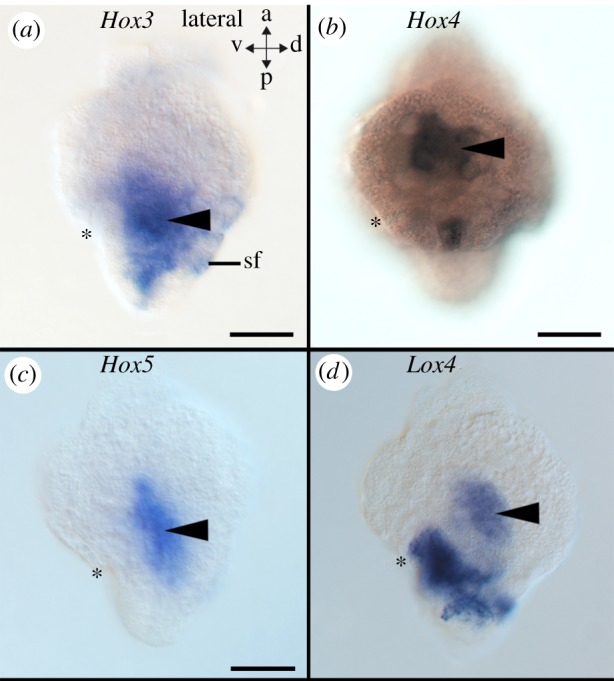
Mesodermal Hox gene expression in the early-stage trochophore larva of the scaphopod *Antalis entalis*. The mouth is labelled with an asterisk and all views are lateral with the ventral side facing to the left. *Hox3* (*a*), *Hox4* (*b*), *Hox5* (*c*) and *Lox4* (*d*) are expressed in internal mesodermal domains (arrowheads) that are located between the endoderm and ectoderm. sf, shell field. Scale bars, 50 µm.

**Figure 3.**
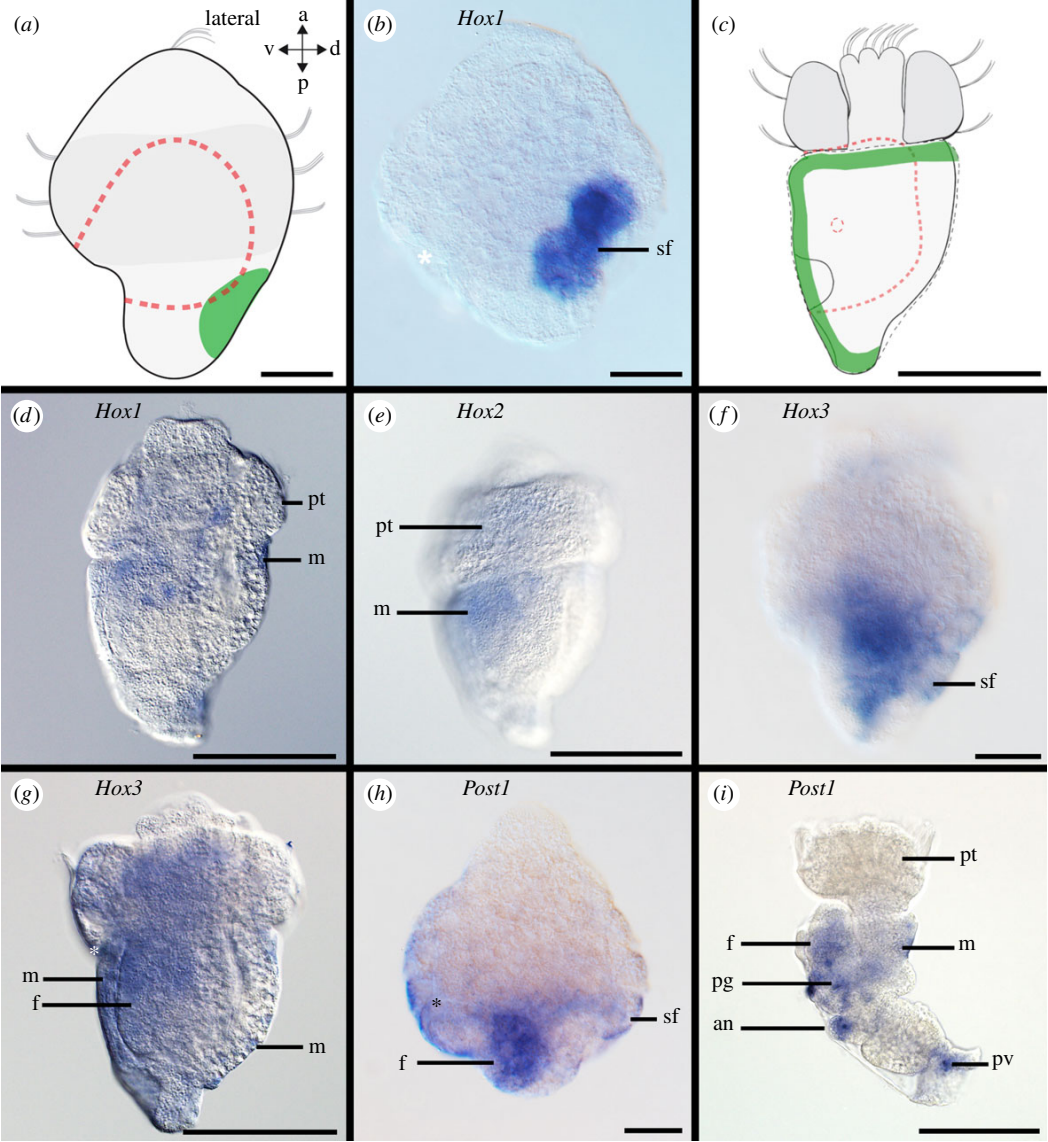
Hox gene expression in the scaphopod shell field. Blastopore and mouth are labelled with asterisks in differential interference contrast images. All lateral views. (*a*) Sketch of early-stage trochophore with shell field (green), digestive tract (dashed red line), and prototroch (dark grey). (*b,d*) *Hox1* expression in shell field (sf) and shell-secreting mantle margin (m) of early-stage (*b*) and early mid-stage trochophore (*d*). (*c*) Sketch of an early mid-stage trochophore with the shell-secreting mantle margin (green), digestive track (dashed red line) and prototroch (dark grey). (*e*) *Hox2* expression in the mantle of an early mid-stage trochophore. (*f,g*) *Hox3* expression in the shell field and mesoderm of an early-stage trochophore (*f*) and in the mantle and foot of an early mid-stage trochophore (*g*). (*h,i*) *Post1* expression in foot and shell field of early-stage trochophore (*h*) and the foot, mantle, pedal ganglia (pg), pavilion (pv), and anus (an) of late mid-stage trochophore (*i*). pt, prototroch. Scale bars, 50 µm.

**Figure 4.**
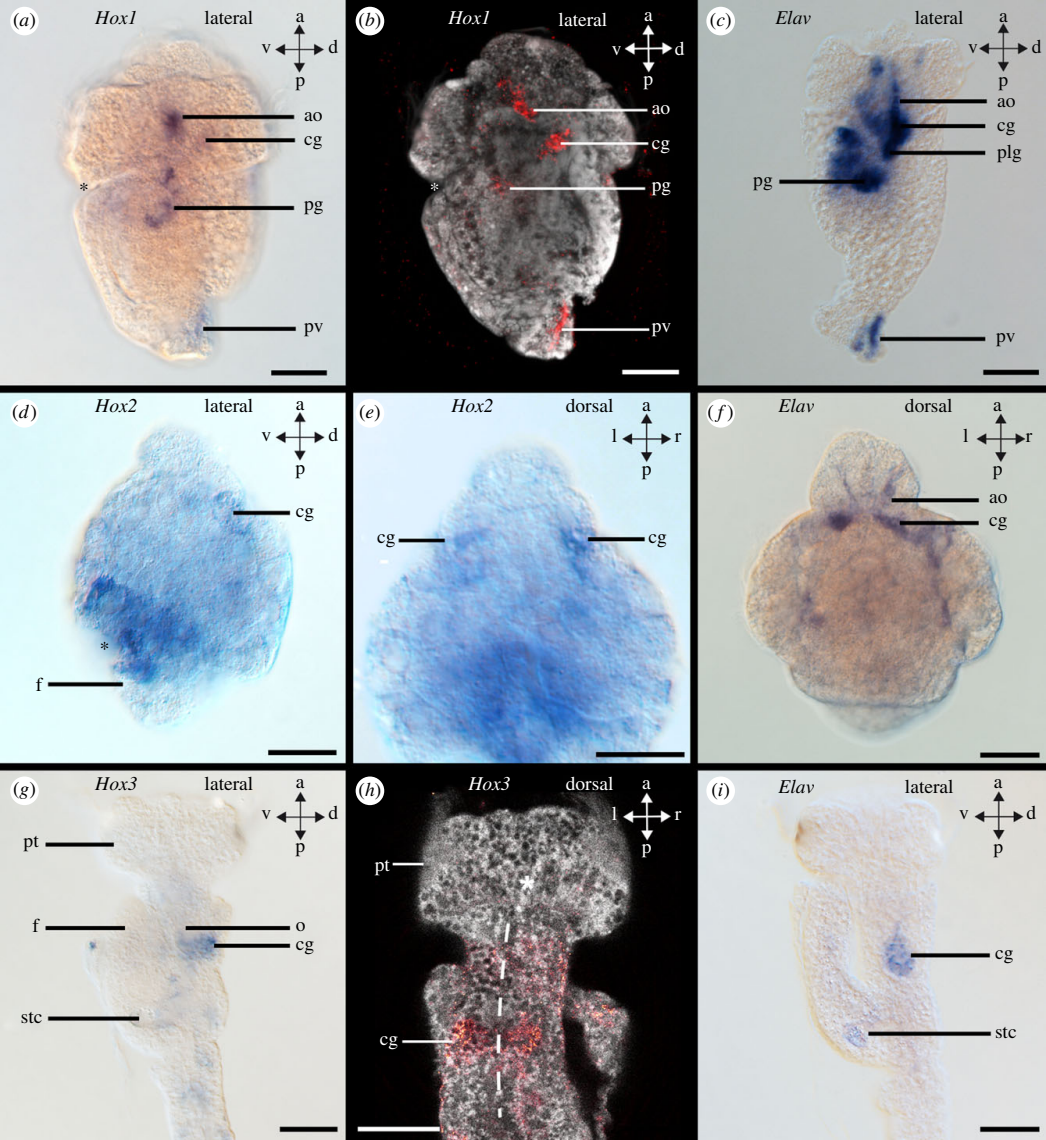
Hox gene expression in the scaphopod nervous system. Blastopore and mouth are labelled with asterisks. Differential interference contrast image (DIC) of Hox gene expression (left column), confocal reflection scan of Hox expression (red) (middle column), DIC image of *Elav* expression in developing neurons (right column). (*a,b*) *Hox1* expression in the anlagen of the cerebral (cg) and pedal ganglia (pg), cells of the apical organ (ao), and the pavilion (pv) of an early mid-stage trochophore. (*d,e*) *Hox2* expression in the anlagen of the cerebral ganglia (cerebral pits). (*g,h*) *Hox3* expression in the cerebral ganglia of a late-stage trochophore. (*h*) Dashed line marks digestive tract. dt, digestive tract; o, oesophagus; f, foot; plg, pleural ganglia; pt, prototroch; stc, statocyst. Scale bars: 50 µm.

**Figure 5.**
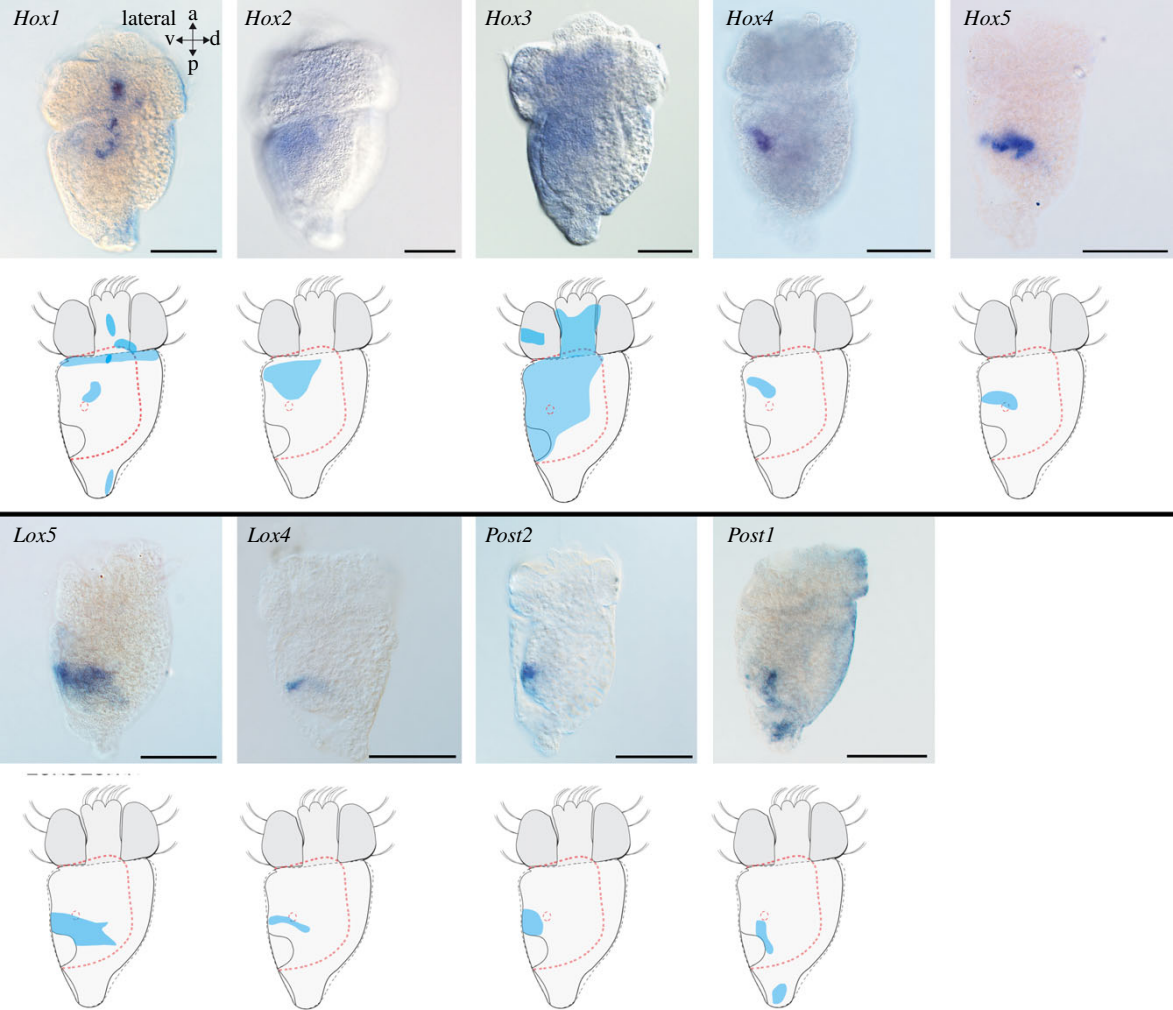
Expression of Hox genes in the early mid-stage trochophore of the scaphopod *Antalis entalis*. Differential interference contrast images (first and third rows) of Hox gene expression and sketches as summary of expression (second and fourth rows). Digestive tract (dashed red lines) and statocysts (dashed red circles) are indicated. All lateral views and ventral faces to the left. Hox genes are expressed in a near-to staggered fashion in the scaphopod's mid-stage trochophore with only *Hox3* and *Post2* deviating from this pattern. Note the weak unspecific staining on the prototroch and mantle (*Post1*). Scale bars, 100 µm.

### (b) Early-stage trochophore larva

Hox and ParaHox genes are expressed predominately in the foot (*Hox2, Hox3, Lox5, Lox4, Post1, Post2*), the shell field (*Hox1, Hox3, Post1*), around the mouth (*Hox2, Hox4*), in the mesoderm (*Hox3, Hox4, Hox5, Lox4*), the pavilion (*Post1, Post2*), and in the region of the hindgut (*Hox3*, *Lox5*, *Cdx*) (figures 2–4; electronic supplementary material, figures S3A-C, S5A-C, S7A-C, S9A-C, S11A-C, S13A-C, S15A-C, S17A-C, S19A-C, S21A-C). In particular, *Hox2* is expressed in the cerebral pits, which are *Elav*-expressing bilateral invaginations close to the apical organ that give rise to the cerebral ganglia (figure 4*d–f*; electronic supplementary material, figure S1A-D; for the location of cerebral pits, see [20]). *Elav* (*embryonic lethal, abnormal visual system*) is a marker of nascent neurons [21,22]. In addition, the nervous system of early-stage trochophore larvae as revealed by *Elav*-expression is composed of an apical organ, pedal and pleural ganglia, as well as neurons in a ganglion associated with the prospective posterior mantle opening (pavilion) (electronic supplementary material, figure S1A-D; [5]).

### (c) Early mid-stage trochophore larva

**Figure 6.**
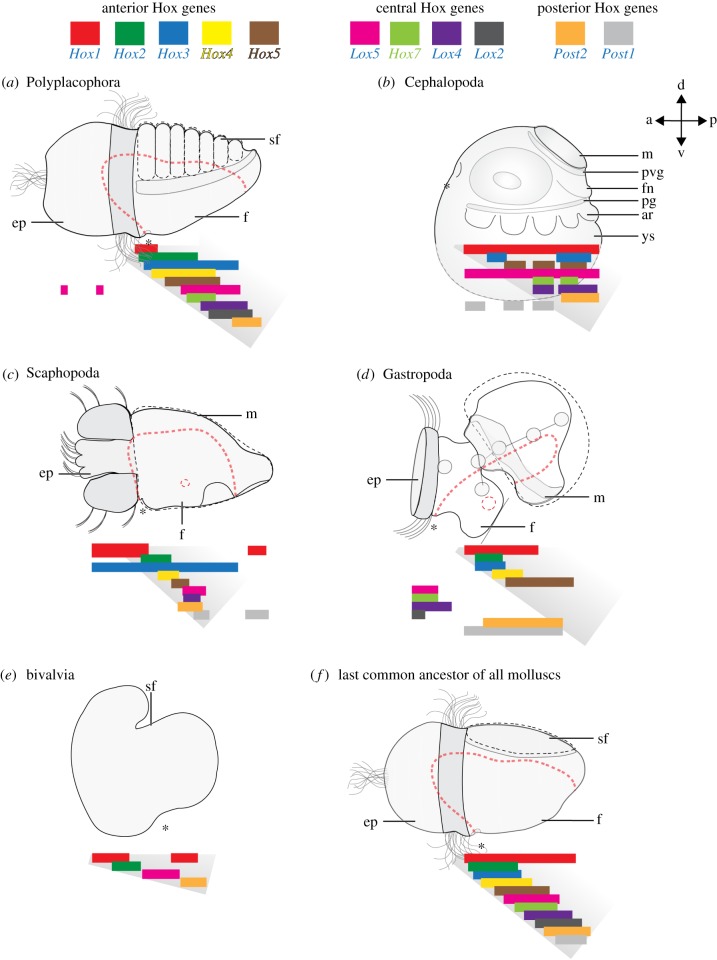
Traces of staggered Hox expression in molluscan developmental stages. Coloured bars indicate anterior–posterior extension of individual Hox gene expression domains and underlying grey shading highlights genes that are expressed in a staggered fashion. Digestive tract (stippled red lines), shell/shell plates (dashed black line), blastopore/mouth (asterisks) and prototroch/velum (shaded in dark grey) are indicated. (*a*) Late-stage trochophore of the polyplacophoran *Acanthochitona crinita*. (*b*) Stage 19/20 embryo of the cephalopod *Euprymna scolopes*. (*c*) Early mid-stage trochophore of the scaphopod *Antalis entalis*. (*d*) Pre-torsional veliger larva of the gastropod *Gibbula varia*. (*e*) Gastrula of the bivalve *Patinopecten yessoensis*. (*f*) Inferred trochophore larva of the last common molluscan ancestor. a, anterior; ar, arm; d, dorsal; ep, episphere; f, foot; fn, funnel; m, mantle; v, ventral; p, posterior; pg, pedal ganglion; pvg, palliovisceral ganglion; sf, shell field; ys, yolk sac.

Hox genes are expressed in a near-to staggered and partially overlapping fashion along the larval anterior–posterior axis (figures 5 and 6). *Hox1* is co-expressed with *Elav* in the anlagen of the cerebral and pedal ganglia, cells of the apical organ, the anterior-most region of the foot and the pavilion (figure 5; electronic supplementary material, figures S1E-H; S3D-F). In addition, the anterior-most mantle margin expresses *Hox1* (electronic supplementary material, figure S3D-F). The anterior-lateral ventral portion of the foot and the anterior mantle margin are *Hox2* expression domains (figure 5; electronic supplementary material, figure S5D-F). *Hox3* is expressed in lateral and ventral portions as well as in some dorsal portions of the mantle, in addition to the lateral–ventral trochoblasts and two lateral apical cells (figure 5; electronic supplementary material, figure S7D-F). Weak *Hox3* expression is also present in the anterior and median region of the foot. *Hox4* is expressed in the pedal ganglia and on the level of the statocysts, while *Hox5* is expressed by cells on the level of the statocysts and slightly more posterior to the latter (figure 5; electronic supplementary material, figures S9D-F, S11D-F). The *Lox5* expression domain extends posterior-laterally from the statocysts to the posterior-most region of the foot (figure 5; electronic supplementary material, figure S13D-F). *Lox4* expressing cells are present ventrally to the statocysts and slightly more posterior to the latter (figure 5; electronic supplementary material, figure S15D-F). The posterior foot and the pavilion express *Post1*, while a domain extending from anterior to posterior to the statocysts, including the pedal ganglia, expresses *Post2* (figure 5; electronic supplementary material, figures S17D-F, S19D-F). *Cdx* is expressed in the region of the hindgut (electronic supplementary material, figure S21D-F).

### (d) Late mid-stage trochophore larva

*Hox1* is expressed in the cerebral and pedal ganglia, the anterior mantle opening, and the pavilion. *Hox2* is expressed in the anterior mantle margin, slightly more posterior to the *Hox1* expression domain, in the foot, and in the pedal ganglia (electronic supplementary material, figures S2A-D, S4A-C, S6A-C). The *Hox3* expression domain is located in the anterior-median portion and in dorsal parts of the foot (electronic supplementary material, figure S8A-C). *Hox4* is expressed in the pedal ganglia anterior to the statocysts, while *Hox5* expression is located laterally in the foot on the level of the statocysts and anterior to the latter in two spots more medially and ventrally than the other *Hox5* expression domain (electronic supplementary material, figures S10A-C, S12A-C). *Lox5* expression is situated lateral, anterior and posterior to the statocysts in the foot and *Lox4* is expressed in two spot-like domains anterior to the statocysts and in two domains posterior to the statocysts in the foot (electronic supplementary material, figures S14A-C, S16A-C). *Post1* is expressed in the pavilion, two pedal domains anterior to the statocysts, and posterior to the latter (electronic supplementary material, figure S18A-C). In addition, a *Post1* expression domain is present in the anterior-lateral and dorsal mantle margin and close to the anus. *Post2* is expressed anterior to the statocysts in the foot and posterior to the statocysts with additional expression in the pavilion (electronic supplementary material, figure S20A-C). *Cdx* expression is only present in the posterior digestive tract similar to the early mid-stage trochophore (electronic supplementary material, figure S22A-C).

### (e) Late-stage trochophore larva

*Hox1* is expressed in the anterior mantle margin, the cerebral ganglia and probably in the palliovisceral ganglia (electronic supplementary material, figures S2E-H; S4D-F). Co-expression of Hox genes and *Elav* only occurs in the cerebral ganglia that are the only domains of the nervous system that express *Elav* during this larval stage. *Hox2* expression domains are located close to the midgut gland, in the lateral mantle and in the region of the cerebral and visceral ganglia (electronic supplementary material, figure S6D-F). *Hox3* is expressed in the cerebral and pedal ganglia (figure 4*g–i*), while *Hox4* is expressed in the region of the visceral ganglia in late-stage trochophores (electronic supplementary material, figures S8D-F, S10D-F). *Hox5* is expressed in the pedal and cerebral ganglia and the *Lox5* expression domain is located between the pedal and the cerebral ganglia (electronic supplementary material, figures S12D-F, S14D-F). *Lox4* is expressed faintly around the statocysts (electronic supplementary material, figure S16D-F). The cerebral ganglia and the anterolateral mantle margin express *Post1*, while *Post2* is expressed in domains anterior and posterior to the statocysts and the visceral mass (electronic supplementary material, figures S18D-F, S20D-F). *Cdx* is expressed in the hindgut and in the foot (electronic supplementary material, figure S22D-F).

### (f) Postmetamorphic/settled individuals

Settled animals express *Hox2*, *Hox3* and *Hox5* in portions of the mantle (electronic supplementary material, figures S6G-I, S8-G-I, S12G-I). *Hox4* is expressed in both captacula anlagen, while *Hox5* is expressed in the pedal ganglia (electronic supplementary material, figures S10G-I, S12G-I). Besides the pedal ganglia and the pavilion ganglion all other major ganglia such as the cerebropleural ganglia, the putative visceral ganglia, and bilateral lateral domains on the level between foot and intestine express *Elav* (figure 2*i–l*; electronic supplementary material, figure S2I-L). *Lox5* is expressed in the anterior foot and *Lox4* is expressed in the buccal region (electronic supplementary material, figures S14G-I, S16G-I). *Post1* and *Post2* are expressed in the anterior portion of the visceral mass, while *Hox3*, *Hox4*, *Hox5*, *Lox5*, *Lox4*, *Post1* and *Post2* are also expressed in the central portion of the visceral mass, probably in the region of the visceral ganglia (electronic supplementary material, figures S8G-I, S10G-I, S12G-I, S14G-I, S16G-I, S18G-I, S20G-I). *Post2* is also expressed dorsally to the statocysts in the posterior region of the foot (electronic supplementary material, figure S20G-I). *Cdx* is expressed in the foot and in the region of the hindgut (electronic supplementary material, figure S22G-I).

## 4. Discussion

### (a) Traces of staggered Hox gene expression in molluscs

Until recently Hox genes have been thought to be expressed in a non-colinear fashion in the Mollusca as demonstrated for the gastropods *Haliotis asinina, Gibbula varia* and the cephalopod *Euprymna scolopes* [7,9–11]. All three conchiferan species have been reported to express Hox genes in distinct morphological structures rather than staggered along the anterior–posterior body axis as it is the case in the majority of ecdysozoans, deuterostomes, and lophotrochozoans investigated [23–33]. A recent study on the polyplacophoran *Ac. crinita*, however, revealed a staggered Hox expression in the trochophore larva of this aculiferan [16,17]. Moreover, another study indicated that staggered expression may also be found in the gastrula stage of a bivalve [34].

Our study demonstrates that the scaphopod *An. entalis* exhibits traces of staggered Hox expression during ontogeny. Seven of nine Hox genes are expressed in a staggered fashion along the larval anterior–posterior axis in early mid-stage trochophores (figures 5 and 6). The anterior-most expression domains are the anlagen of the cerebral ganglia, the apical organ and the anterior-most mantle margin that both express *Hox1* (figure 5). While *Hox2* is expressed slightly more posterior to *Hox1* in the mantle margin, *Hox2*, *Hox4, Hox5, Lox5, Lox4* and *Post1* are predominantly expressed in the foot (figure 5). *Hox3* clearly deviates from the staggered condition with expression domains in the trochoblasts, the foot, two lateral apical cells and large portions of the mantle (figures 5 and 6). In addition, *Post2* expression is not in accordance with the staggered expression pattern because its pedal expression domain is located slightly more anterior to the one of *Lox4* (figures 5 and 6). Notably, all anterior-most *Hox3* expression domains of early mid-stage trochophores that deviate from the staggered expression, such as the trochoblasts and the apical cells, are in ‘larval’ structures that do not contribute to the prospective adult body plan. In the majority of bilaterians, Hox gene expression domains only comprise anlagen of ‘adult’ structures [25–33]. With exception of the annelid *Platynereis dumerilii* that expresses *Hox1* in the larval episphere (same in *An. entalis*), molluscs constitute, so far, the only bilaterians that express Hox genes also in purely larval structures that do not persist through metamorphosis [28]. For instance, the gastropod *G. varia* expresses *Lox5, Hox7, Lox4* and *Lox2* in the velum or in the larval episphere, while only *Lox5* is expressed in the episphere of the polyplacophoran *Ac. crinita* [7,11,16,17].

In contrast to all other developmental stages of the scaphopod *An. entalis*, late mid-stage trochophores also show traces of staggered Hox gene expression, although during this stage *Lox5* and *Post1* (and not *Hox3* and *Post2* as in early mid-stage trochophores) disrupt staggered Hox expression (electronic supplementary material, figures S4A-C, S6A-C, S8A-C, S10A-C, S12A-C, S14A-C, S16A-C, S18A-C, S20A-C). Contrary to early mid-stage trochophores, late mid-stage trochophores do not exhibit any Hox expression in larval structures.

Interestingly, a closer look at the cephalopod *E. scolopes* indicates that all studied Hox genes except for *Lox5* and *Post1* are expressed in a staggered fashion in the stage 19/20 embryo, the earliest developmental stage that exhibits distinct anlagen of all major organ system such as the eyes, mantle, funnel and arm crown (figure 6*b*; [10]). Cephalopod Hox genes are typically expressed in the arm crown with additional expression domains in the funnel, the pedal ganglia, the palliovisceral ganglia or the light organ [10]. Morphological studies suggest that the cephalopod arm crown evolved from the anterior portion of the foot, while the funnel is probably a derivative of the posterior foot ([35–38]; but see [39–41] for a different view). Bearing this topology in mind, *Hox1, Hox3, Hox5, Hox7, Lox4* and *Post2* are expressed in a staggered fashion from anterior (the first arm) to posterior (the funnel; figure 6*b*). By contrast, earlier and later cephalopod developmental stages do not show obvious traces of staggered Hox expression. Interestingly, *Lox5* deviates from the staggered expression pattern in the cephalopod by being expressed more anteriorly, a condition that is also found in polyplacophorans and gastropods but not in scaphopods (figure 6; [7,10,17]). *Lox5* belongs to one of the six posterior Hox genes that are not expressed in a staggered fashion in the gastropod pre-torsional veliger larvae [7,11]. By contrast, all anterior Hox genes, i.e. *Hox1, Hox2, Hox3, Hox4* and *Hox5* are expressed in a staggered fashion in the mantle and in the pedal and pleural ganglia of the gastropod pre-torsional veliger (figure 6*a–d* of present study; [7,9,11]). Notably, staggered Hox expression is neither found in early gastropod and scaphopod trochophores nor in the early stage 18 cephalopod embryo. Remainders are only present in developmental stages with a well-developed foot (scaphopods and gastropods) or arm crown and the funnel (cephalopods) (present study; [7,9–11]). This may be different in bivalves that (putatively) exhibit staggered Hox expression in the gastrula prior to foot formation [34].

### (b) Conchiferan molluscs express Hox genes in distinct morphological structures

Although traces of a staggered Hox expression are present during the ontogeny of all investigated molluscs, our data on the scaphopod *An. entalis* also support the notion that Hox genes pattern morphological structures in the Conchifera [4,10]. In all scaphopod developmental stages, Hox genes are expressed in distinct features such as the shell field (figure 3*b*: *Hox1*), the foot (figure 3*h*: *Post1*), ganglia (figure 4: *Hox1-3*), as well as in trochoblasts and cells of the apical organ (figure 4*a*: *Hox1*; figure 5: *Hox3*). Hox genes are usually not expressed in the anterior-most part of the adult bilaterian nervous system, i.e. the cerebral ganglia in molluscs [10,42]. Therefore, it is surprising that *Hox1-3* are expressed in the developing scaphopod cerebral ganglia, a condition that has only been reported for very few bilaterian species so far, including the polychaete annelid *Capitella teleta* [29] and the cephalochordate *Branchiostoma lanceolatum* [43].

Although scaphopod Hox genes are predominantly expressed in ectodermal derivatives such as the nervous system and the anlagen of the shell field/the shell secreting mantle margin of progressive developmental stages, there are also endodermal and mesodermal expression domains (figure 2). The mesoderm of scaphopods is of dual origin and derives from the mesentoblasts during gastrulation and progressive development [20,44]. Mesodermal cells that will eventually differentiate, among others, into muscle cells are already located in the region between the developing mantle (ectoderm) and the digestive system (endoderm) in the early-stage trochophore larva (fig. 12 in [20,44]). While *Hox3-5* and *Lox4* are expressed in some parts of the mesoderm of scaphopod early-stage trochophores, no mesodermal Hox expression was observed in progressive developmental stages (figure 2; electronic supplementary material, figures S3-S20). Hox genes have been reported to pattern mesodermal derivatives in a variety of bilaterians including molluscs [7,10,11,16,17,45]. Hox genes are expressed to a lesser degree in mesodermal structures of conchiferans than in polyplacophoran aculiferans. As *An. crinita* and representatives of several other bilaterian phyla express individual Hox genes broadly throughout all three germ layers, it may be assumed that a similar condition was also present in the last common ancestor of all molluscs.

Five out of nine Hox genes are expressed in the scaphopod shell field and the shell-secreting mantle margin of progressive developmental stages (*Hox1-4, Post1*; electronic supplementary material, table S1). While in polyplacophorans nine of 10 Hox genes (i.e. all except for *Hox5*) are expressed in the shell fields, gastropods only express four of 11 Hox genes in their shell field (*Hox1, Hox4, Post2, Post1*) and cephalopods none (electronic supplementary material, table S1). Scaphopods express all nine and polyplacophorans all 10 Hox genes in their foot in contrast to gastropods with only three of the 10 Hox genes showing expression in the foot (*Hox2-4*; electronic supplementary material, table S1). As the cephalopod foot homologues, the arms express all seven Hox genes investigated in *E. scolopes* and the funnel four of the seven Hox genes (electronic supplementary material, table S1). All Hox genes are expressed in the nervous system of polyplacophorans, scaphopods and cephalopods, while in gastropods only three of 10 Hox genes are neural (electronic supplementary material, table S1). The cephalopod arms contain the anlagen of the brachial nerve cords and Hox expression domains in the arms are probably also neuroectodermal [10]. Considering a staggered Hox expression as ancestral for Bilateria, the polyplacophoran anterior–posterior expression pattern is closest to this plesiomorphic condition (electronic supplementary material, table S1; [16,17]). Interestingly, the cephalopod Hox expression profile of the nervous system and the foot homologues resembles that of the scaphopod and polyplacophoran more than that of gastropods (electronic supplementary material, table S1). This suggests that scaphopods have retained Hox genes as players acting in anterior–posterior patterning as opposed to their close conchiferan allies but more similar to their distant aculiferan relatives.

### (c) A last common molluscan ancestor with colinear Hox expression?

For the Mollusca, spatial colinear Hox expression has only been suggested for the gastrula of the bivalve *Patinopecten yessoensis* [34]. No genomic information on the organization of Hox loci has been published for any of the other above-mentioned molluscs for which expression data based on *in situ* hybridization are available. Accordingly, for these species it is impossible to decide whether Hox genes are arranged in a clustered or a non-clustered fashion on the genome. Nevertheless, several molluscan genomes have been sequenced and the one of the patellogastropod *Lottia gigantea* shows a clustered arrangement of the Hox genes [46]. By contrast, the genomes of the bivalves *Crassostrea gigas* and *Pinctada fucata* and that of the cephalopod *Octopus bimaculoides* do not show a continuous Hox cluster [47,48]. Temporal colinearity, i.e. the progressive expression of Hox genes according to their location on the chromosome, is probably not present in the scaphopod *An. entalis* and it has not been reported for any other mollusc so far.

A look at other bilaterians shows that a categorization into ‘colinear’ versus ‘non-colinear’ Hox gene expression patterns is not as clear-cut as it is exemplified by Hox expression of fruit fly or mouse embryos in textbooks [23,24]. The cephalochordate *B. lanceolatum* possesses an intact Hox cluster but no colinear Hox expression [43]. The platyhelminth *Schmidtea mediterranea* and the leech *Helobdella robusta* exhibit signs of staggered Hox expression, however both show a disrupted cluster [49,50]. The polychaete annelid *C. teleta* as well as the brachiopod *Terebratalia transversa* have a split Hox cluster but while the polychaete exhibits staggered Hox expression, this is not the case in the brachiopod [29,33]. If a colinear Hox expression and genomic clustering were ancestral for Bilateria, then the above-mentioned examples show that staggered Hox expression, clustered genomic organization and temporal colinearity would have been lost independently in several lophotrochozoan taxa. This substantiates the notion that scaphopods, cephalopods and gastropods lost colinear Hox expression to varying degrees independently of each other. If true, then Hox genes were not ‘recruited independently for development’ of the cephalopod arm crown and funnel as previously assumed [10], but show a rather conserved function in anterior–posterior patterning of these cephalopod foot homologues. The fact that the Hox cluster of one cephalopod species is disrupted does not necessarily argue against this scenario [48]. This is exemplified by the ascidian *Oikopleura dioica* that exhibits staggered Hox expression while having a disintegrated cluster [26]. Its chordate ancestor, however, presumably showed colinear Hox expression.

## 5. Conclusion

Hox genes are expressed in a near-to staggered fashion in mid-stage trochophore larvae of a conchiferan mollusc, the scaphopod *An. entalis.* This appears as a remainder of the presumably ancestral condition for Bilateria that has been conserved in an aculiferan mollusc, the polyplacophoran *Ac. crinita*. As reported for gastropod and cephalopod conchiferans, Hox genes also pattern distinct morphological structures in scaphopods. Similar to other molluscs and one annelid, Hox genes are also expressed in scaphopod larval structures which is in contrast to other bilaterians. Our results demonstrate that a mosaic of ancestral and novel molecular machinery underlies scaphopod development, a condition that is less evident in other conchiferans which also show traces of staggered Hox expression. Our data show that, during anterior–posterior patterning, scaphopod Hox genes behave more similar to their distant aculiferan relatives than to their close conchiferan allies. A colinear mode of Hox expression in the last common ancestor of Annelida, Nemertea, as well as Mollusca also argues for a last common lophotrochozoan ancestor with colinear Hox expression.

## Supplementary Material

Electronic supplementary material including a detailed Material and method section, Figures and Tables

## Data accessibility

All Hox, ParaHox and *Elav* gene sequences analysed in this study are available on Genbank (www.ncbi.nlm.nih.gov/genbank; accession numbers: KX365088–KX365099; MH107247–MH107249).
