# Supplementary material for: Staggered Hox expression is more widespread among molluscs than previously appreciated
Source: Proc Biol Sci. 2018 Oct 10;285(1888):20181513. doi: 10.1098/rspb.2018.1513 (PMC6191704; doi:10.1098/rspb.2018.1513)
Supplement: Electronic supplementary material including a detailed Material and method section, Figures and Tables [file rspb20181513supp1.pdf]

Electronic Supplementary Material

Staggered Hox expression is more widespread among mollusks than previously appreciated

Tim Wollesen*, Sonia Victoria Rodríguez Monje, André Luiz de Oliveira, and Andreas Wanninger

Department of Integrative Zoology, Faculty of Life Sciences, University of Vienna, Althanstraße 14, 1090 Vienna, Austria. *

Email: Tim Wollesen: tim.wollesen@embl.de

Sonia Victoria Rodríguez Monje: sonia.rodriguez@univie.ac.at

André Luiz de Oliveira: andre.luiz.de.oliveira@univie.ac.at

Andreas Wanninger: andreas.wanninger@univie.ac.at

*Corresponding author: Tim Wollesen; Email: tim.wollesen@embl.de

Current address: European Molecular Biology Laboratory Heidelberg, Meyerhofstr. 1, 69117 Heidelberg, Germany

Orcid ID Wollesen: 0000-0003-0464-1254

Orcid ID Wanninger: 0000-0002-3266-5838

Orcid ID De Oliveira: 0000-0003-3542-4439

Proceedings of the Royal Society B – Biological Sciences

Material and methods: pages 2-4

Results with List of Abbreviations and Figures S1-S24: pages 5-29

Table S1: pages 29-30

References: pages 31-32

Material and methods

Ethics, collection and culture of animals

Adults of the scaphopod *Antalis entalis* Jeffreys 1869 were collected from approximately 30 m depth by the staff of the research vessel Neomys off the coast of Roscoff (France) in the summers 2014 and 2017. Animals spawned and developmental stages were reared at 18-20°C as described previously (Wollesen et al. 2015).

RNA extraction and fixation of animals for *in situ* hybridization experiments

Several hundred individuals of early cleavage stages to settled metamorphosed individuals were investigated. All developmental stages were carefully anesthetized in 7.14% $MgCl_2$ before fixation for *in situ* hybridization experiments or RNA extraction and transcriptome sequencing as previously described (Wollesen et al. 2015; De Oliveira et al. 2016).

Alignment and phylogenetic analysis

A thorough phylogenetic analysis on scaphopod Hox and ParaHox gene sequences was carried out previously (De Oliveira et al. 2016) and the scaphopod Hox and ParaHox gene sequences are published (Accession numbers: KX365088 to KX365099). Nucleotide sequences of *Elav* orthologs were retrieved from the published transcriptomes of the scaphopod *A. entalis*, the bivalve *Nucula tumidula*, and the polyplacophoran *Acanthochitona crinita* by using bilaterian gene orthologs in BLAST searches against the latter transcriptomes (De Oliveira et al. 2016; Accession numbers: MH107247-MH107249). Predicted amino acid sequences of Elav and metazoan Elav orthologs were aligned using MAFFT v7.123b (Katoh and Standley 2014). The edited multiple sequence alignment used for the phylogenetic analysis was obtained with BMGE software with the following parameters: -h 1 –b 1 –m BLOSUM30 –t AA (Figure S23; Criscuolo and Gribaldo 2010). The maximum likelihood phylogenetic analysis was carried out with Phyml v3.0 (Guindon et al. 2010)

including the predicted amino acid sequences of cloned *Elav* and metazoan orthologs, with JTT model of amino acid replacement, estimated with Prottest3 v3.4.2. (Darriba et al. 2011), gamma-distributed rates, and 1000 non-parametric bootstrap replicates. The phylogenetic tree was manually rooted using FigTree v1.4.1 (Figure S24; Rambaut 2008).

Molecular isolation of RNA transcripts

A first strand cDNA Synthesis Kit for rt-PCR (Roche Diagnostics GmbH, Mannheim, Germany) was used for first-strand cDNA synthesis of the RNA pooled from different developmental stages of *Antalis entalis.* Identified *Elav*, Hox and ParaHox gene orthologs were used to design gene-specific primers and PCR products were size-fractioned by gel electrophoresis, gel bands of the expected lengths were excised and cleaned up using a QIAquick Gel Extraction Kit (QIAgen, Hilden, Germany). By insertion into pGEM-T Easy Vectors (Promega, Mannheim, Germany) cleaned-up products were cloned. Plasmid minipreps were grown overnight, cleaned-up with the QIAprep Spin MiniprepKit (QIAgen), and sent off for sequencing.

Probe synthesis and whole-mount *in situ* hybridization

Riboprobe templates were amplified via standard PCR from miniprep plasmids using M13 forward and reverse primers as described previously (Wollesen et al. 2015). *In vitro* transcription reactions were performed with these templates, digoxigenin-UTP (DIG RNA Labeling Kit, Roche Diagnostics), and SP6/ T7 polymerase (Roche Diagnostics GmbH) for the syntheses of antisense riboprobes, according to the manufacturer's instructions. In whole-mount *in situ* hybridization experiments, specimens were rehydrated into PBT (phosphate buffered saline + 0.1% Tween-20) and treated with Proteinase-K at 37°C for 10 min (20 µg/ml in PBT). Specimens were pre-hybridized in hybridization buffer for 4-10 h at 55-60°C. Hybridization was performed at the same temperature with probe concentrations ranging between 0.5 and 1 µg/ ml for 21-24 h. A DIG-labeled AP-antibody was used at a dilution of 1:5000 to 1:2500 in blocking solution at 4°C over night. Color development in the NBT/ BCIP/ Alkaline Phosphatase buffer solution took 6-24 hrs at

4°C. A minimum of 30 individuals per stage was investigated. The majority of whole-mount preparations was cleared in a solution of benzyl-benzoate and benzyl alcohol, mounted on objective slides and analyzed. Preparations were documented with an Olympus BX53 Microscope (Olympus, Hamburg, Germany). In addition, developmental stages were scanned with a Leica confocal SP5 II microscope (Leica Microsystems, Wetzlar, Germany) using bright-field, autofluorescence, and reflection mode scans to understand the precise location of transcripts (Jekely and Arendt 2007). If necessary, images were processed with Adobe Photoshop 9.0.2 software (San Jose, CA, USA) to adjust for contrast and brightness. Sketch drawings were created with Adobe Illustrator CC 2015.1.0 (Adobe Systems, Inc., San Jose, CA; USA).

Results:

Table List of abbreviations for Figures S1-22

**Abbreviation**	**Term**	**Abbreviation**	**Term**
a	anterior	m	mantle
an	anus	mg	midgut
ao	apical organ	mgg	midgutgland
apc	cell of apical organ	p	posterior
cg	cerebral ganglion	pg	pedal ganglion
cpi	cerebral pit	plg	pleural ganglion
cpg	cerebropleural ganglion	pt	prototroch
cpt	captacula	pv	pavilion
d	dorsal	r	right
dg	digestive gland	sf	shell field
e	esophagus	stc	statocyst
ed	endoderm	tb	trochoblast
f	foot	v	ventral
hg	hindgut	vg	visceral ganglion
int	intestine	vm	visceral mass
l	left		

The following figures show dorsal, ventral, and lateral views of scaphopod developmental stages. Notably, the respective panels do not necessarily depict the dorsal-most, ventral-most or lateral-most views but rather highlight more dorsal, ventral, or lateral aspects of gene expression that are of relevance to this work.

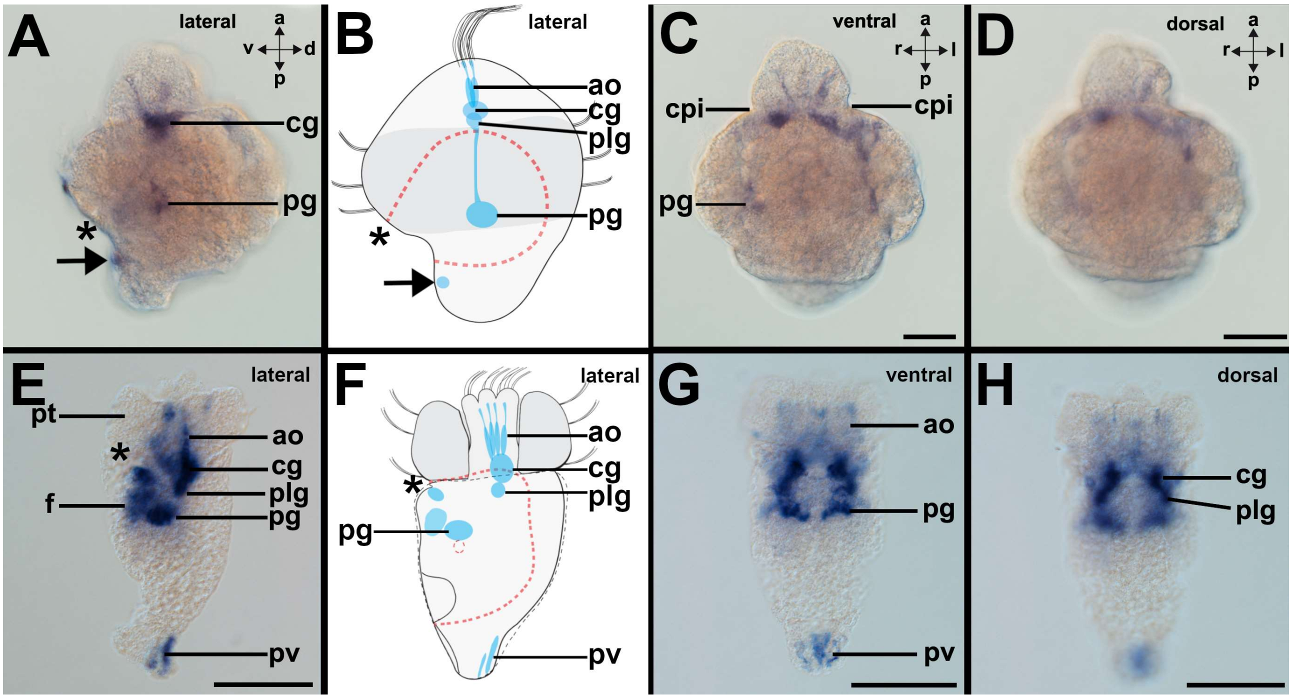

Figure S1. *Elav* gene expression during early development of the scaphopod *Antalis entalis.* The mouth is labeled with an asterisk. A-D: Early-stage trochophores express *Elav* in their nascent cerebral ganglia below the cerebral pits. Additional *Elav* expressing nerve cells are present in the apical organ (arrow), the putative pedal and pleural ganglia as well as in the region posterior to the anus (arrow). E-H (same orientation as in A-D): All major ganglia of early mid-stage trochophores such as the cerebral, pleural and pedal ganglia as well as cells of the apical organ and the posterior mantle opening (pavilion) express *Elav* in early mid-stage trochophore larvae. Scale bars: 100 µm.

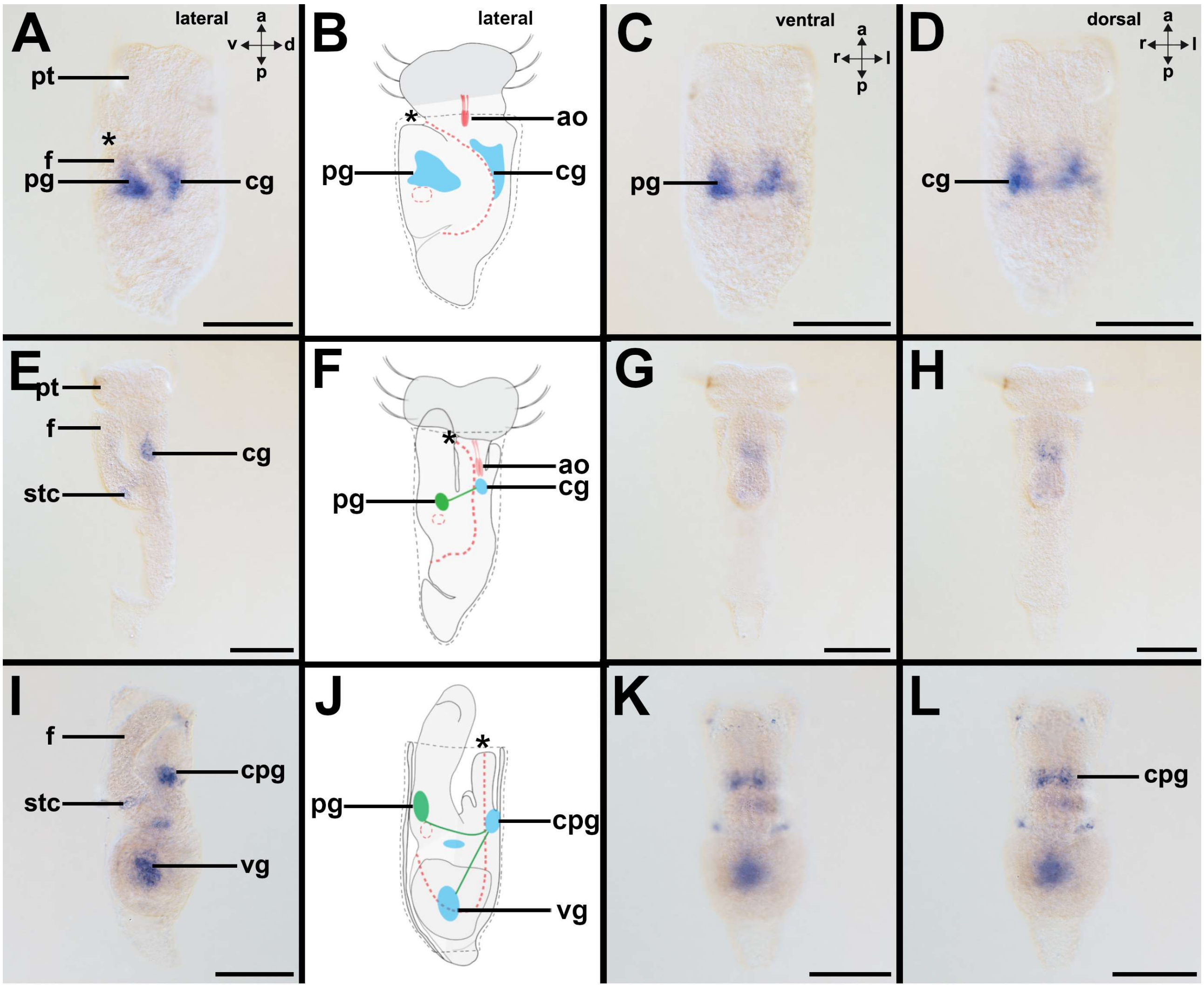

Figure S2. *Elav* gene expression during late development of the scaphopod *Antalis entalis.*

The mouth is labeled with an asterisk. A-D: Late mid-stage trochophores express *Elav* in their pedal and cerebral ganglia. E-H (same orientation as in A-D): Late-stage trochophores express *Elav* in their cerebral ganglia. I-L (same orientation as in A-D): Postmetamorphic individuals possess *Elav* nerve cells in the cerebropleural ganglia, in addition to *Elav* nerve cells in bilaterial lateral domains on the level between foot and intestines. Scale bars: 100 µm.

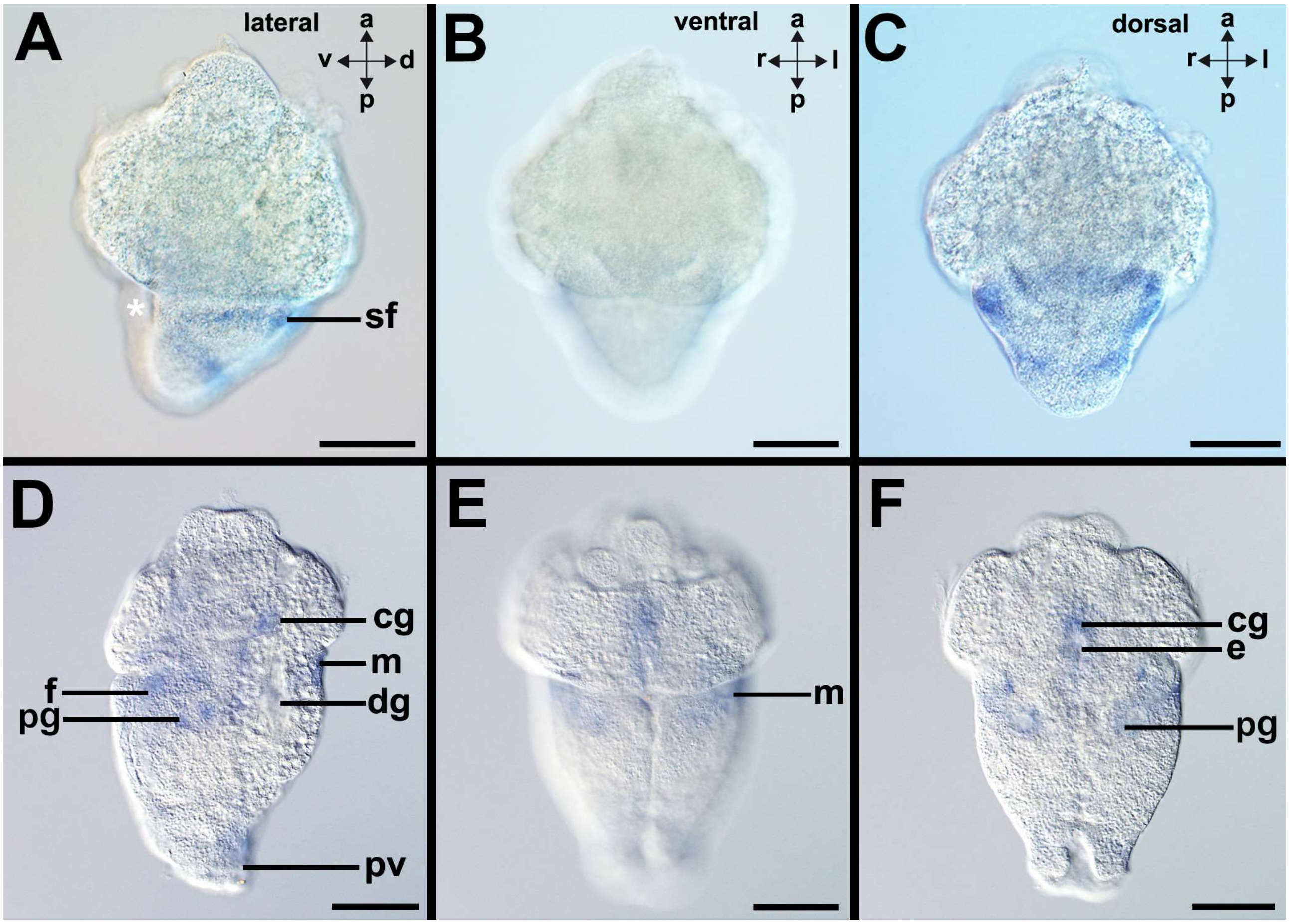

Figure S3. *Hox1* gene expression during early development of the scaphopod *Antalis entalis.* The mouth is labeled with an asterisk. A-C: Early-stage trochophores express *Hox1* in their shell field. D-F (same orientation as in A-C): Early mid-stage trochophores express *Hox1* in the anlagen of the cerebral ganglion, the anterior-most region of the foot, the anterior-most mantle margin, and the pavilion. Scale bars: 100 µm.

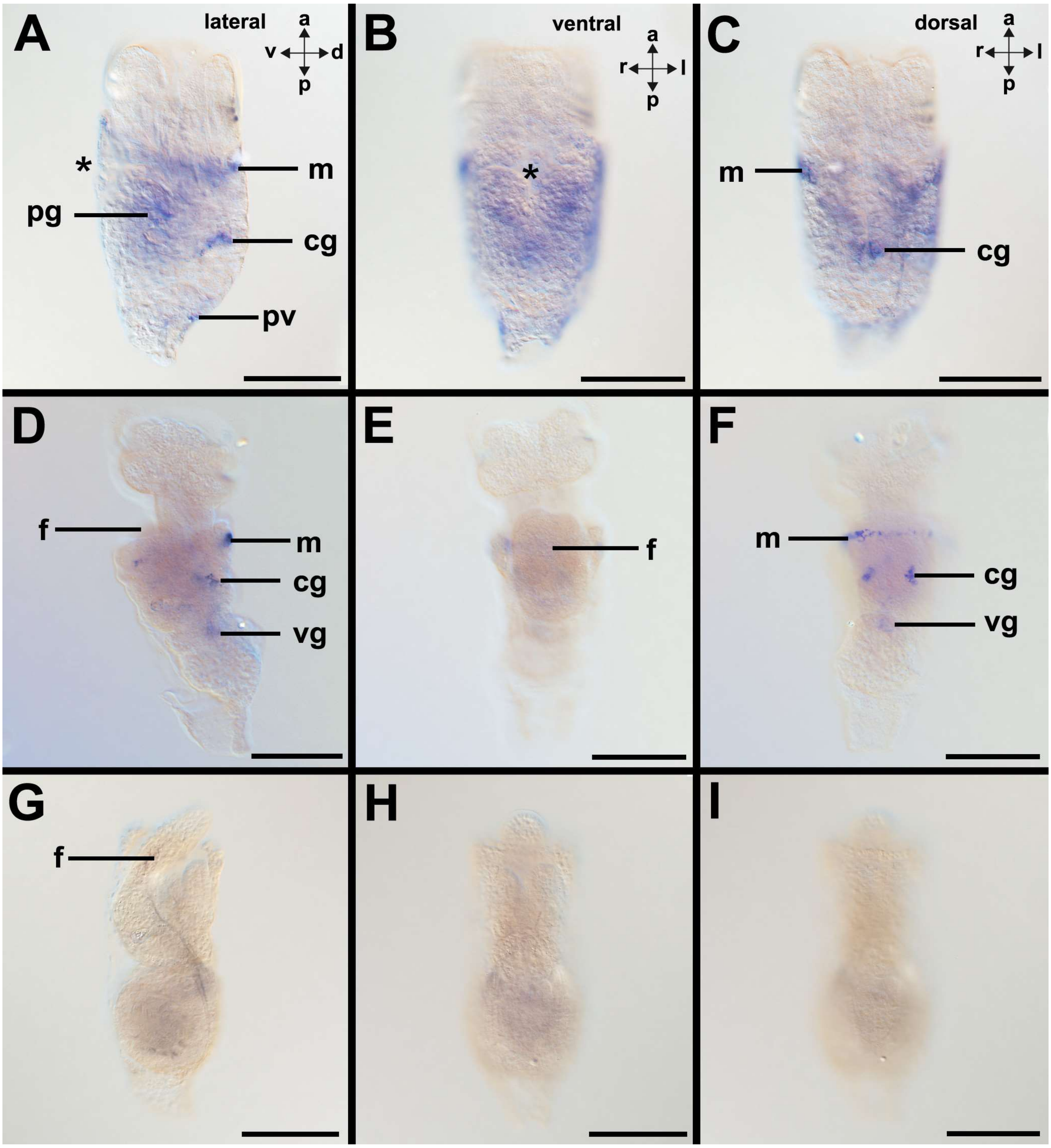

Figure S4. ***Hox1*** **gene expression during late development of the scaphopod** ***Antalis entalis.***

The mouth is labeled with an asterisk. A-C: Late mid-stage trochophores express *Hox1* in the cerebral and pedal ganglion, the anterior mantle opening, and the pavilion. D-F (same orientation as in A-C): Late-stage trochophores express *Hox1* in the anterior mantle margin, the cerebral ganglia, and probably the visceral ganglia. G-I (same orientation as in A-C): No expression of *Hox1* has been detected in postmetamorphic individuals. Scale bars: 100 µm.

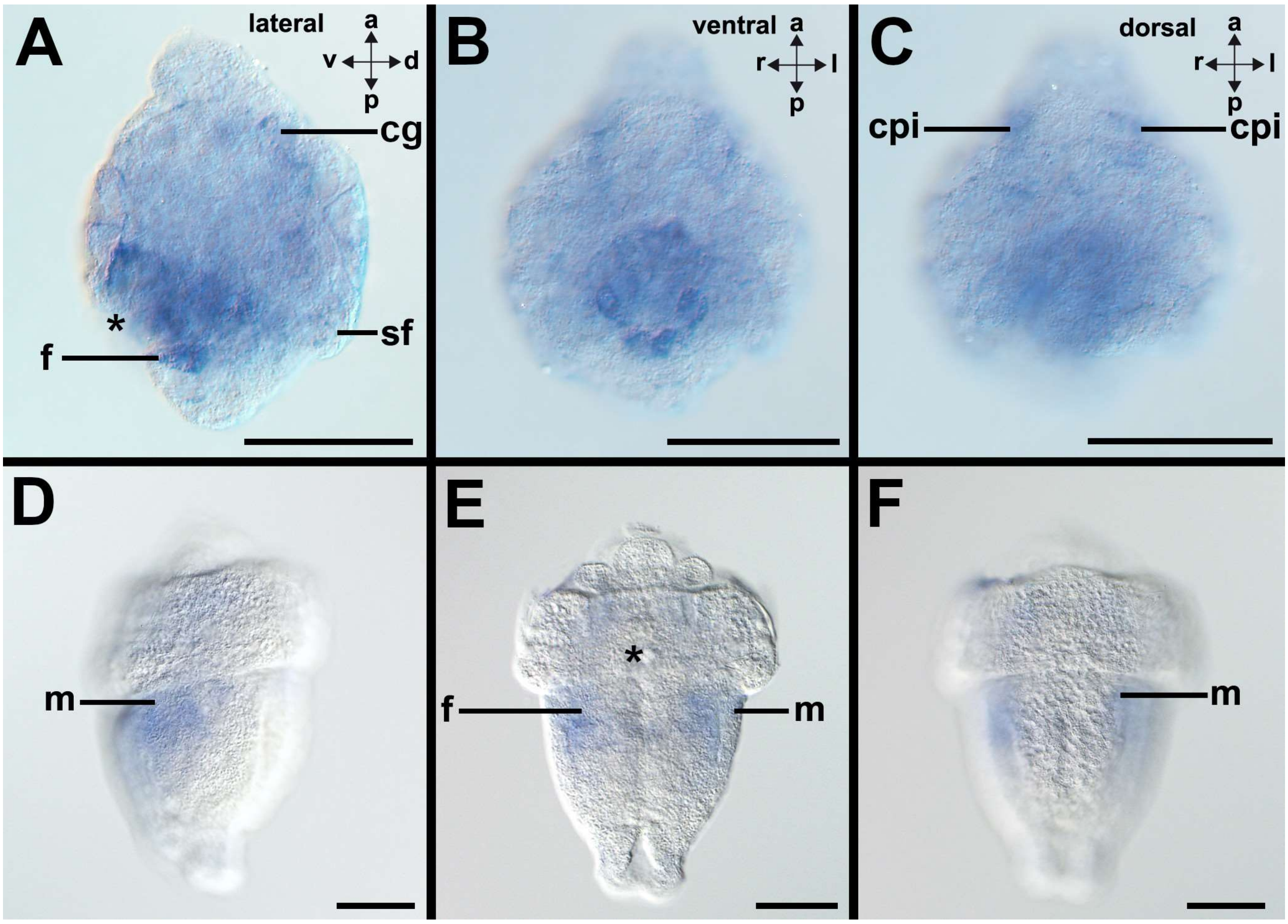

Figure S5. ***Hox2*** **gene expression during early development of the scaphopod** ***Antalis entalis.*** The mouth is labeled with an asterisk. A-C: Early-stage trochophores express *Hox2* in the foot, the nascent cerebral ganglia located in the cerebral pits, and around the mouth. D-F (same orientation as in A-C): Early mid-stage trochophores express *Hox2* in the anteriolateral ventral foot portion (shown in E) and the anterior mantle margin (shown in D and F). Scale bars: 100 µm.

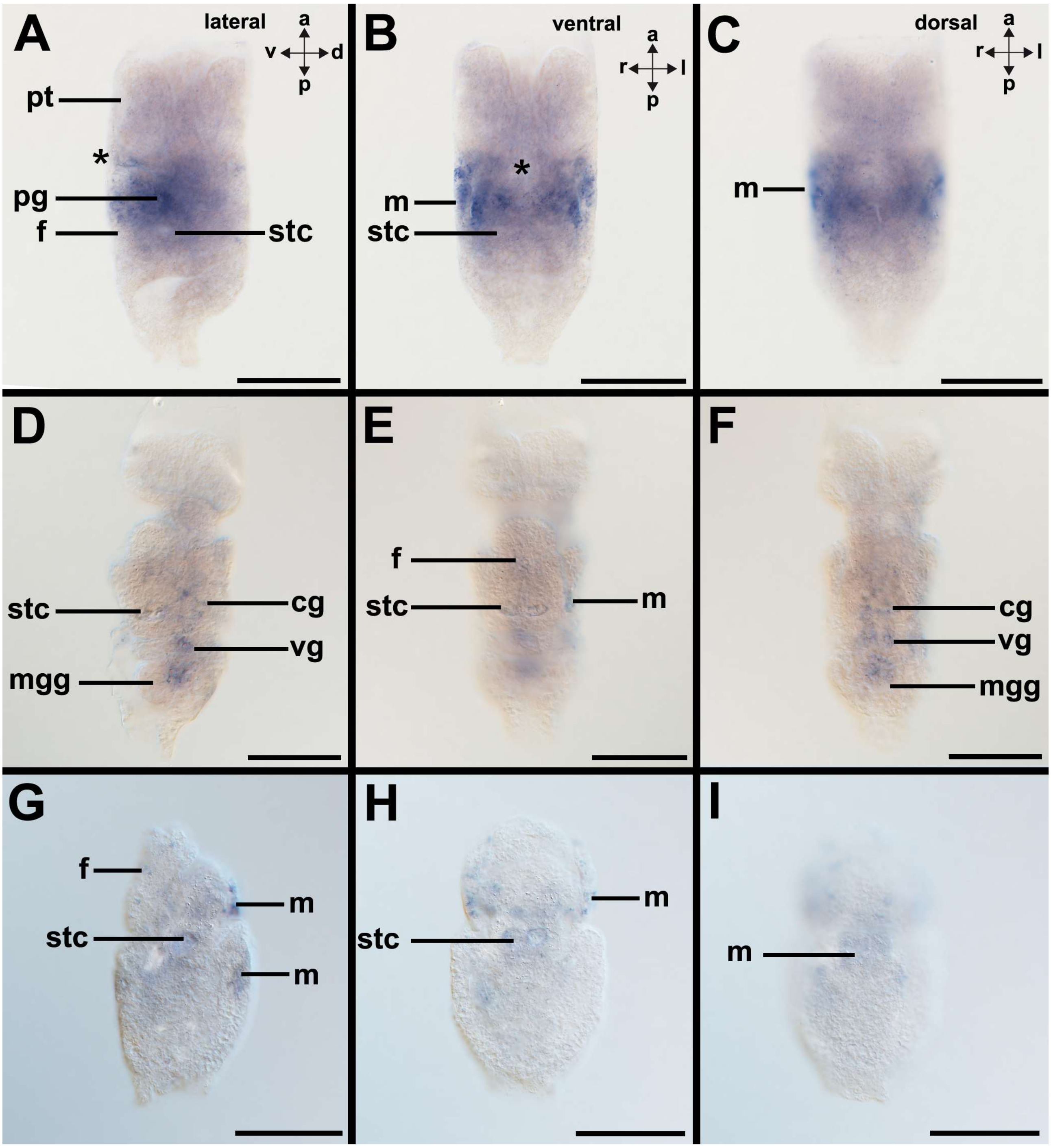

Figure S6. *Hox2* gene expression during late development of the scaphopod *Antalis entalis.*

The mouth is labeled with an asterisk. A-C: Late mid-stage trochophores express *Hox2* in the anterior mantle margin, slightly more posterior to the *Hox1* expression domain, in the foot, and in the pedal ganglia. D-F (same orientation as in A-C): Late-stage trochophores express *Hox2* close to the midgut gland, in the lateral mantle, and in the region of the cerebral and visceral ganglia. G-I (same orientation as in A-C): Postmetamorphic individuals express *Hox2* in dorsal and lateral

portions of the mantle. Scale bars: 100 μm.

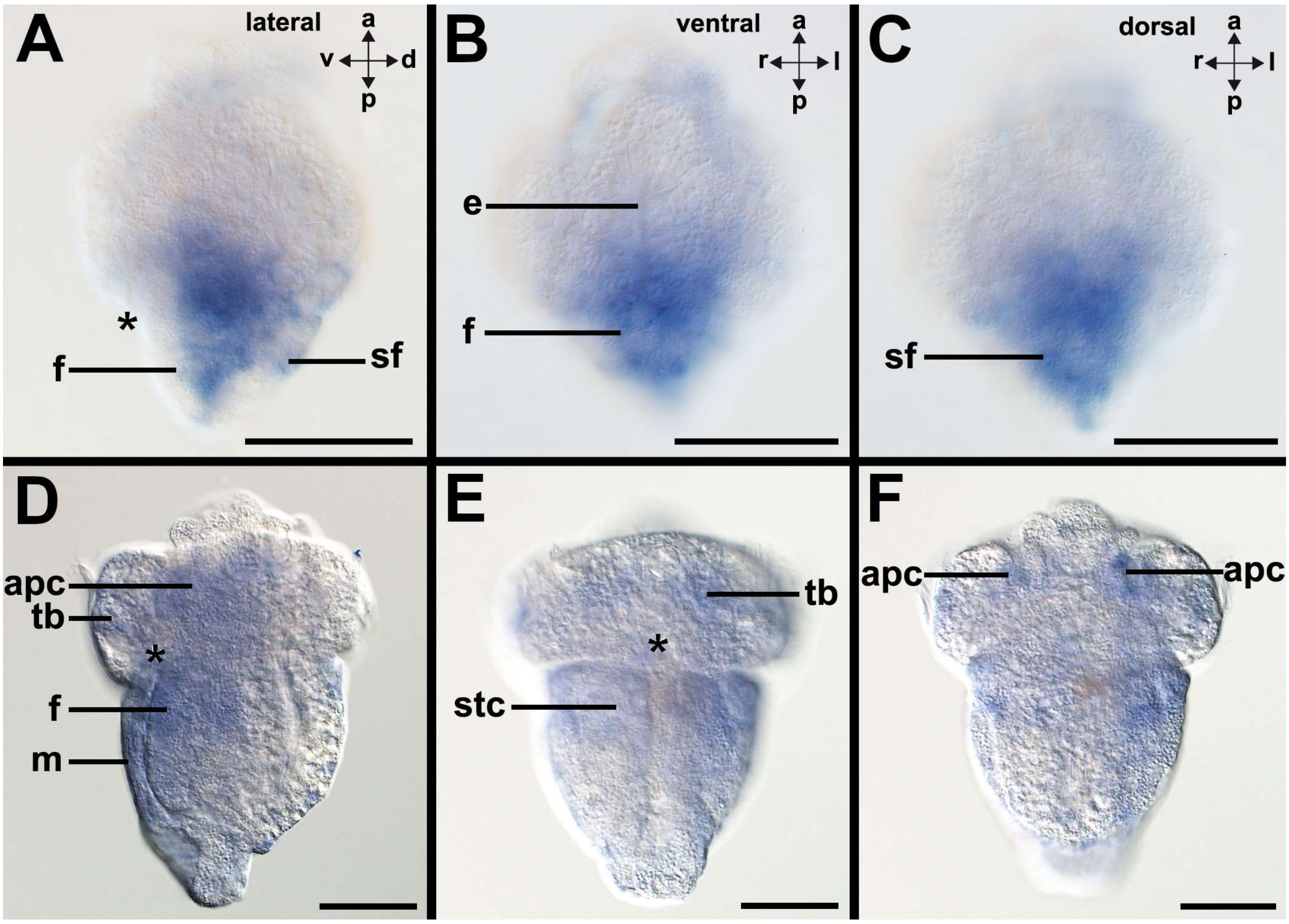

Figure S7. ***Hox3*** **gene expression during early development of the scaphopod** ***Antalis entalis.*** The mouth is labeled with an asterisk. A-C: Early-stage trochophores express *Hox3* in the foot, shell field, and mesoderm. D-F (same orientation as in A-C): Early mid-stage trochophores express *Hox3* in lateral and ventral portions as well as some dorsal portions of the mantle, in addition to the ventral trochoblasts and two lateral apical cells. Weak *Hox3* expression is also present in the anterior and median region of the foot. Scale bars: 100 μm.

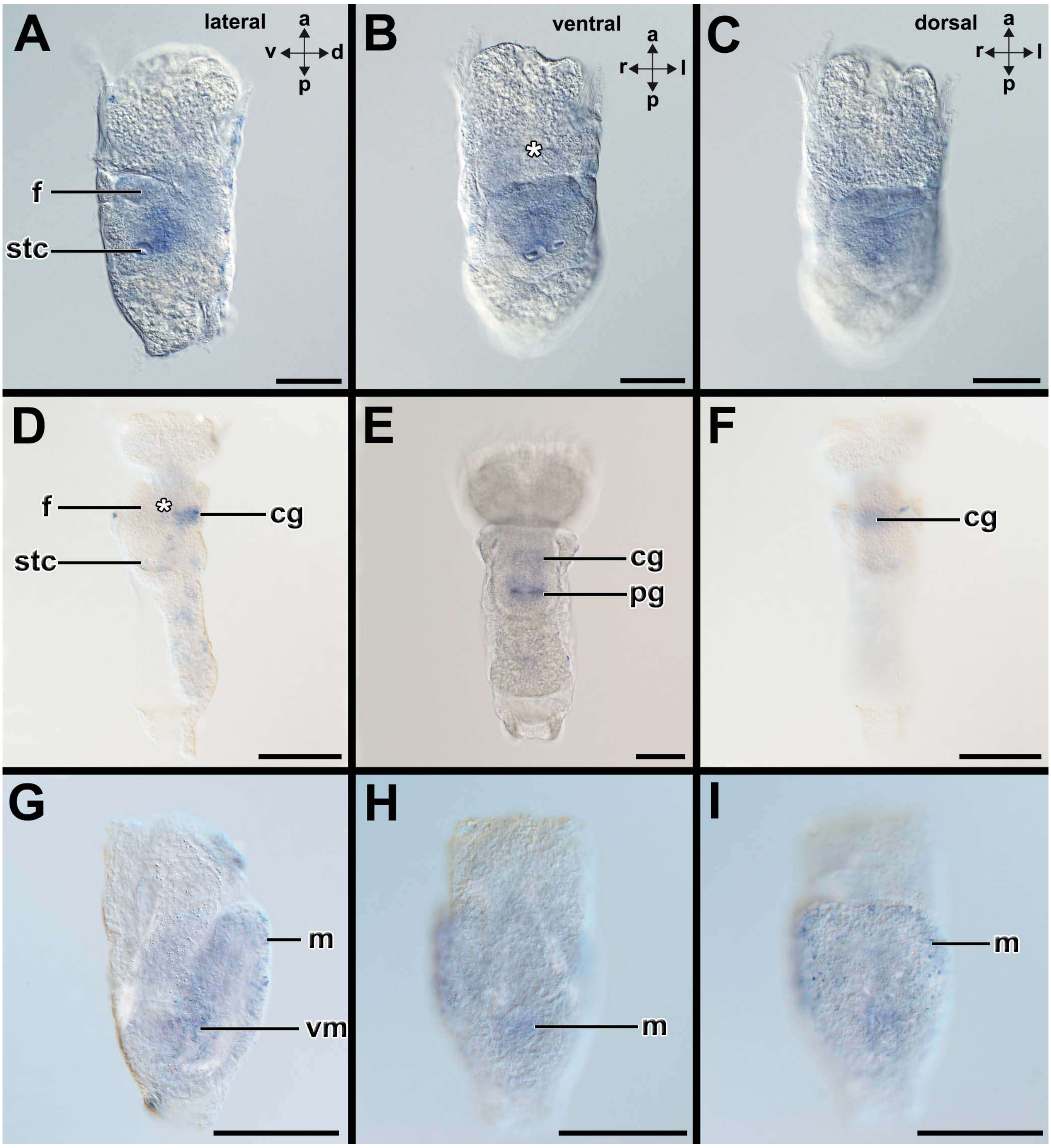

Figure S8. ***Hox3*** **gene expression during late development of the scaphopod** ***Antalis entalis.***

The mouth is labeled with an asterisk. A-C: Late mid-stage trochophores express *Hox3* in the anteromedian portion and in dorsal parts of the foot. D-F (same orientation as in A-C): Late-stage trochophores express *Hox3* in the cerebral and pedal ganglia. G-I (same orientation as in A-C): Postmetamorphic individuals express *Hox3* in portions of the mantle. Scale bars: 100 µm.

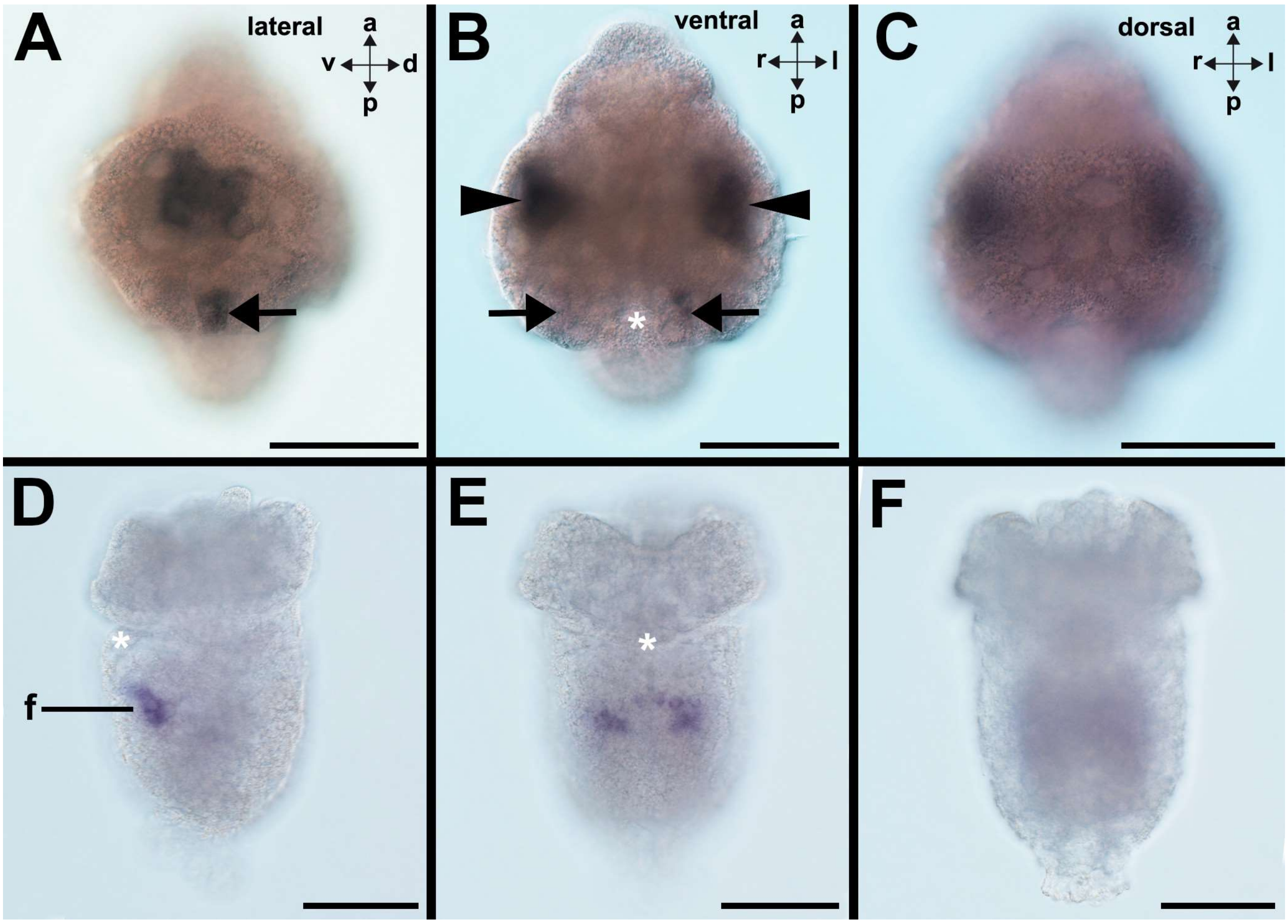

Figure S9. ***Hox4*** **gene expression during early development of the scaphopod** ***Antalis entalis.*** The mouth is labeled with an asterisk. A-C: Early-stage trochophores express *Hox4* around the mouth (arrows) and laterally in a domain below the trochoblasts (arrowheads). D-F (same orientation as in A-C): Early mid-stage trochophores express *Hox4* in the pedal ganglia and on the level of the statocysts. Scale bars: 100 µm.

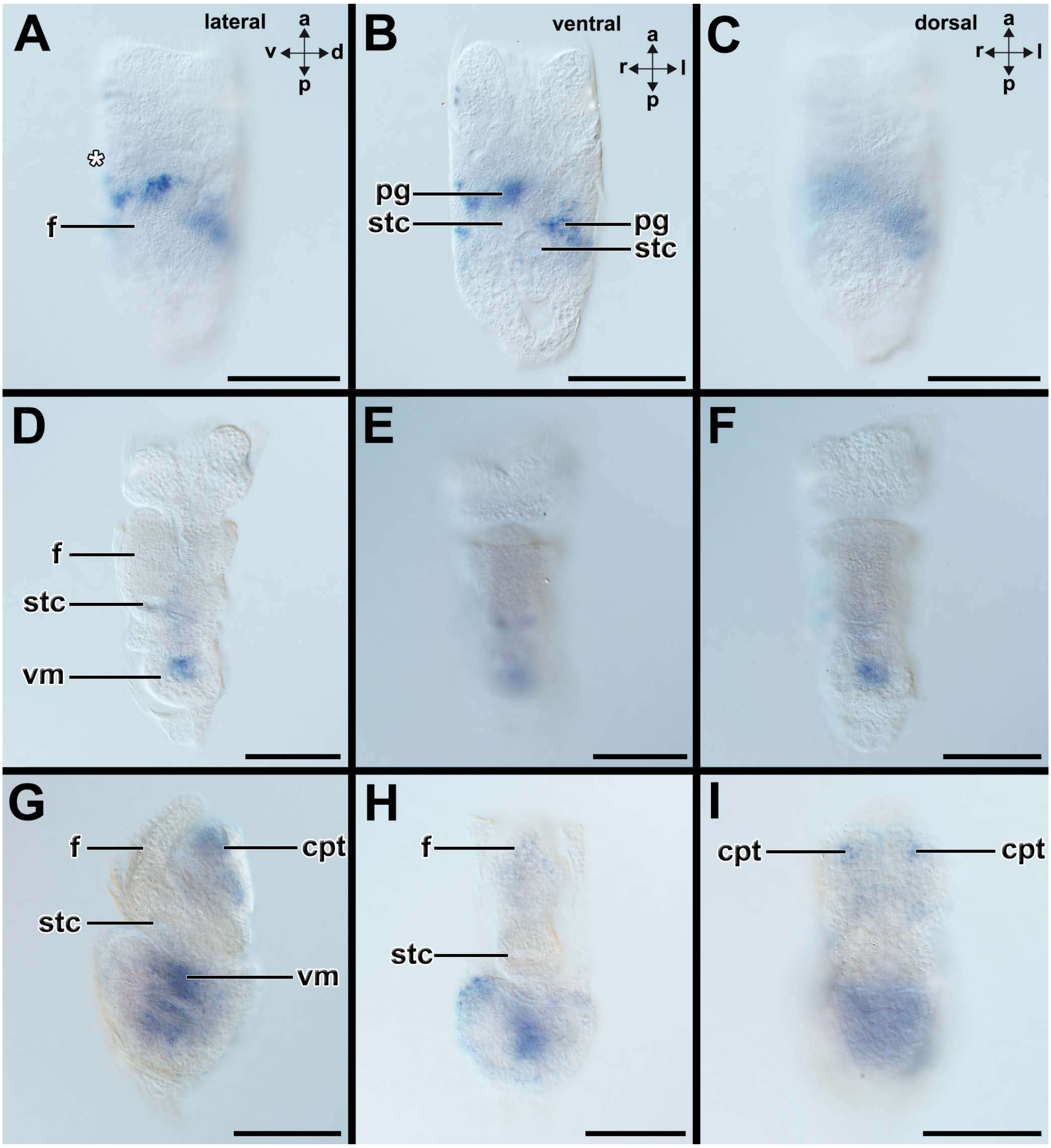

Figure S10. ***Hox4*** **gene expression during late development of the scaphopod** ***Antalis entalis.*** The mouth is labeled with an asterisk. A-C: Late mid-stage trochophores express *Hox4* in the pedal ganglia anterior to the statocysts. D-F (same orientation as in A-C): Late-stage trochophores do not express *Hox4*. G-I (same orientation as in A-C): Postmetamorphic individuals express *Hox4* in both captacula buds. Scale bars: 100 µm.

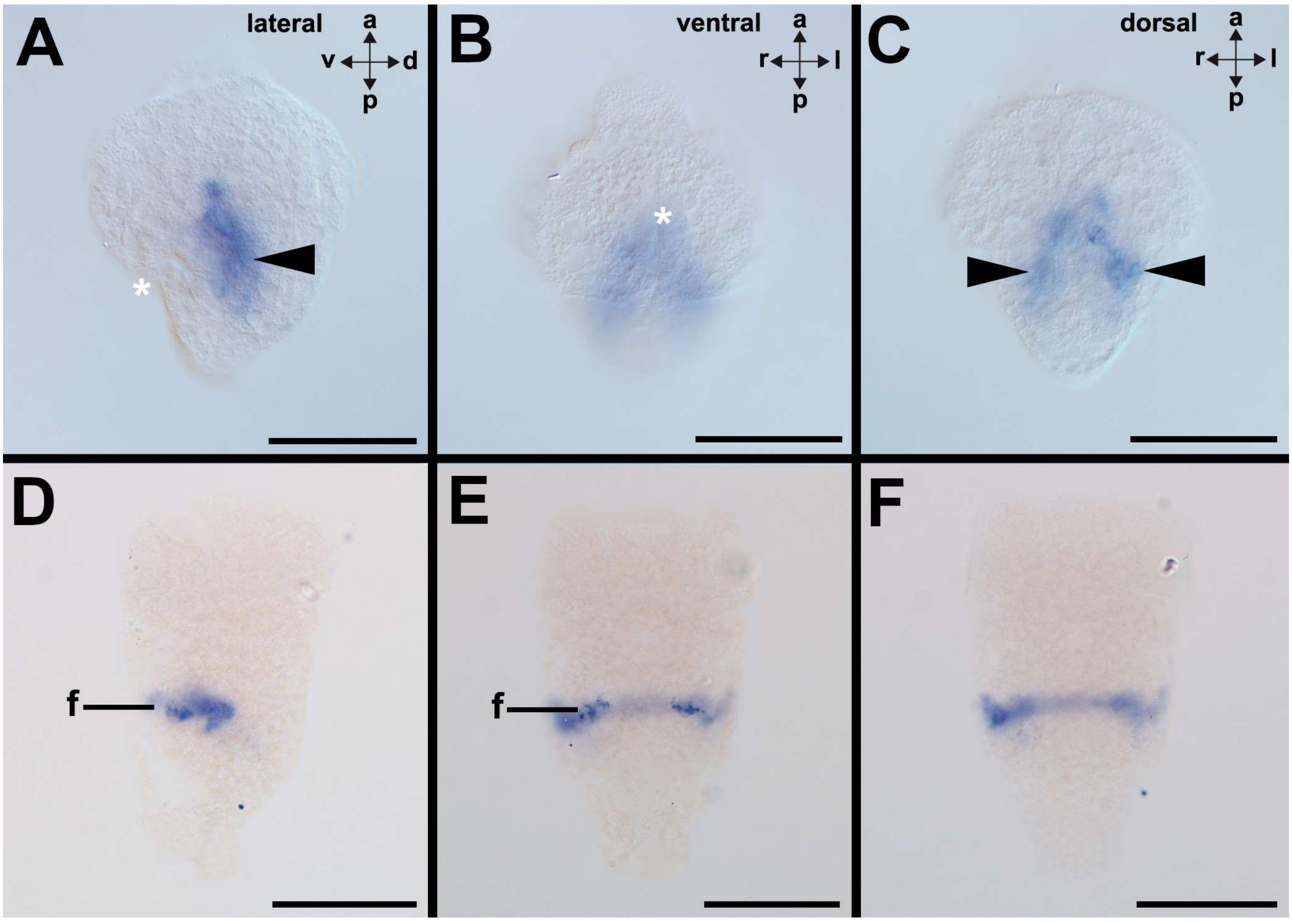

Figure S11. ***Hox5*** **gene expression during early development of the scaphopod** ***Antalis entalis.*** The mouth is labeled with an asterisk. A-C: Early-stage trochophores express *Hox5* in a larger interior region (arrowheads). D-F (same orientation as in A-C): Early mid-stage trochophores express *Hox5* in cells on the level of the statocysts and slightly more posterior to the latter in the foot. Scale bars: 100 µm.

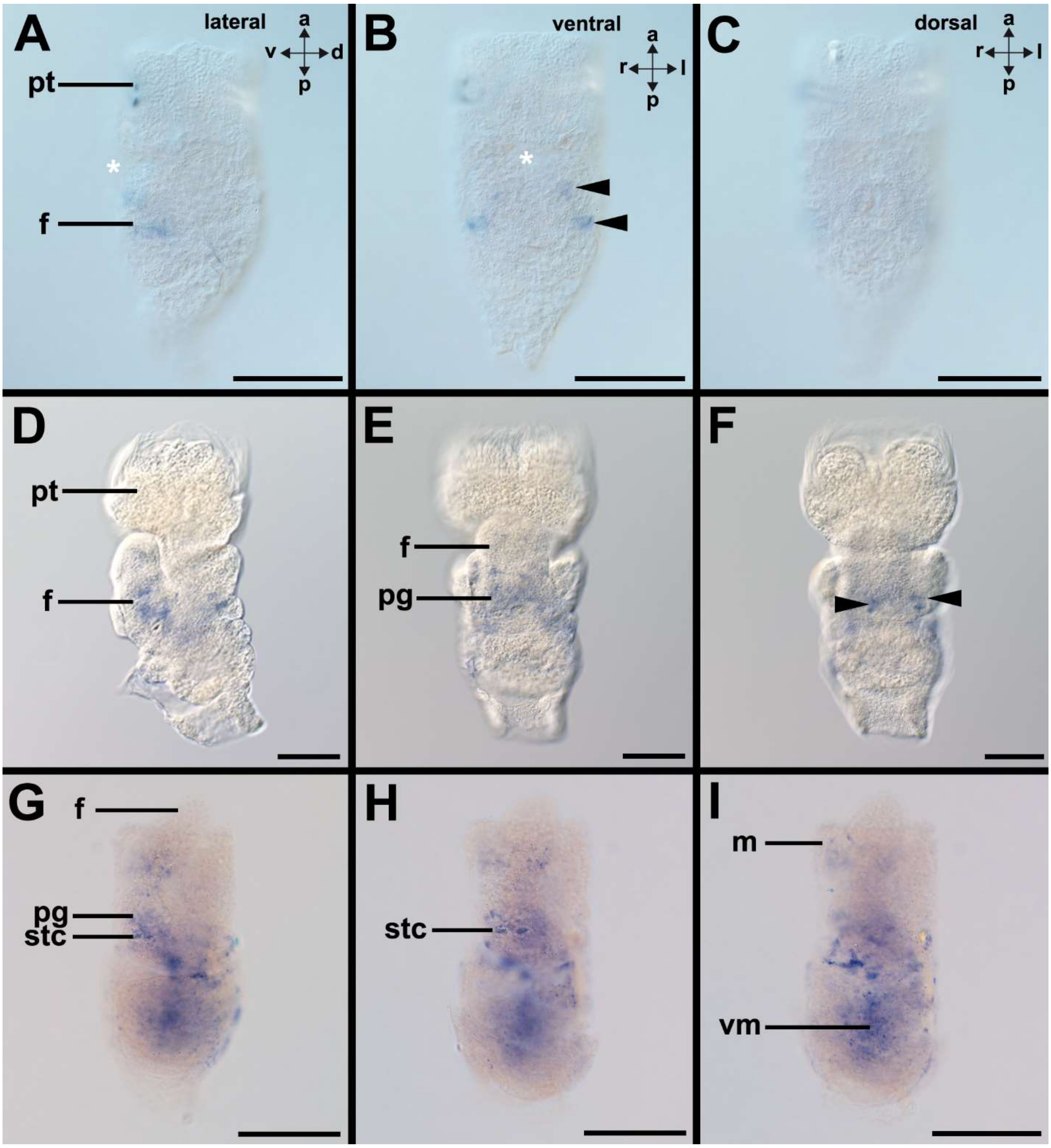

Figure S12. ***Hox5*** **gene expression during late development of the scaphopod** ***Antalis entalis.*** The mouth is labeled with an asterisk. A-C: Late mid-stage trochophores express *Hox5* in the foot on the level of the statocysts and anterior to the latter in two spots more medially and ventrally than the other *Hox5* expression domain. D-F (same orientation as in A-C): Late-stage trochophores express *Hox5* in the pedal and cerebral ganglia. G-I (same orientation as in A-C): Postmetamorphic individuals express *Hox5* in the pedal ganglia. Scale bars: 100 µm.

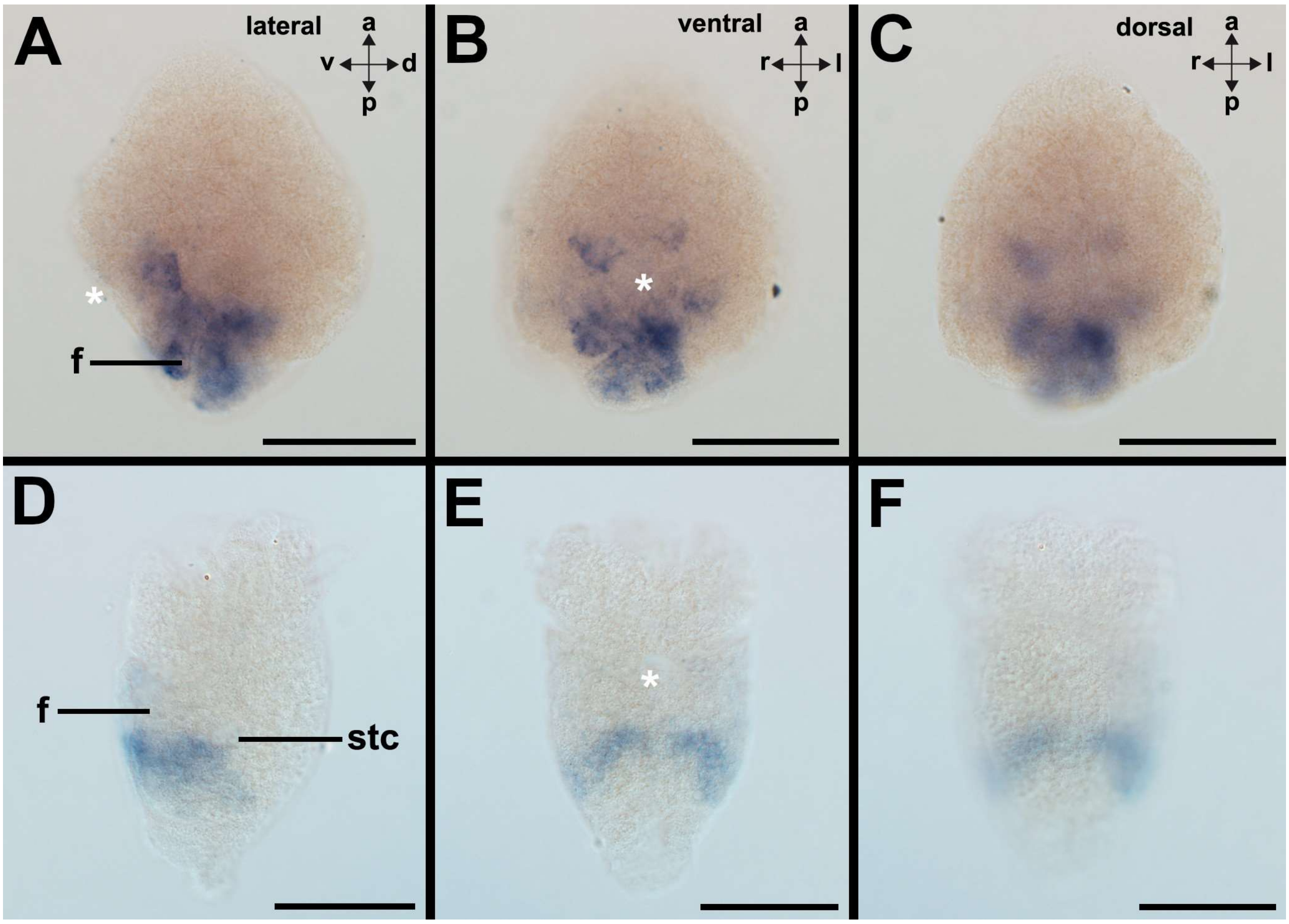

Figure S13. ***Lox5/ Hox6*** **gene expression during early development of the scaphopod** ***Antalis entalis.*** The mouth is labeled with an asterisk. A-C: Early-stage trochophores express *Lox5* in the foot anlage. D-F (same orientation as in A-C): The *Lox5* expression domain of early mid-stage trochophores extends posterolaterally from the statocysts to the posterior-most foot. Scale bars: 100 µm.

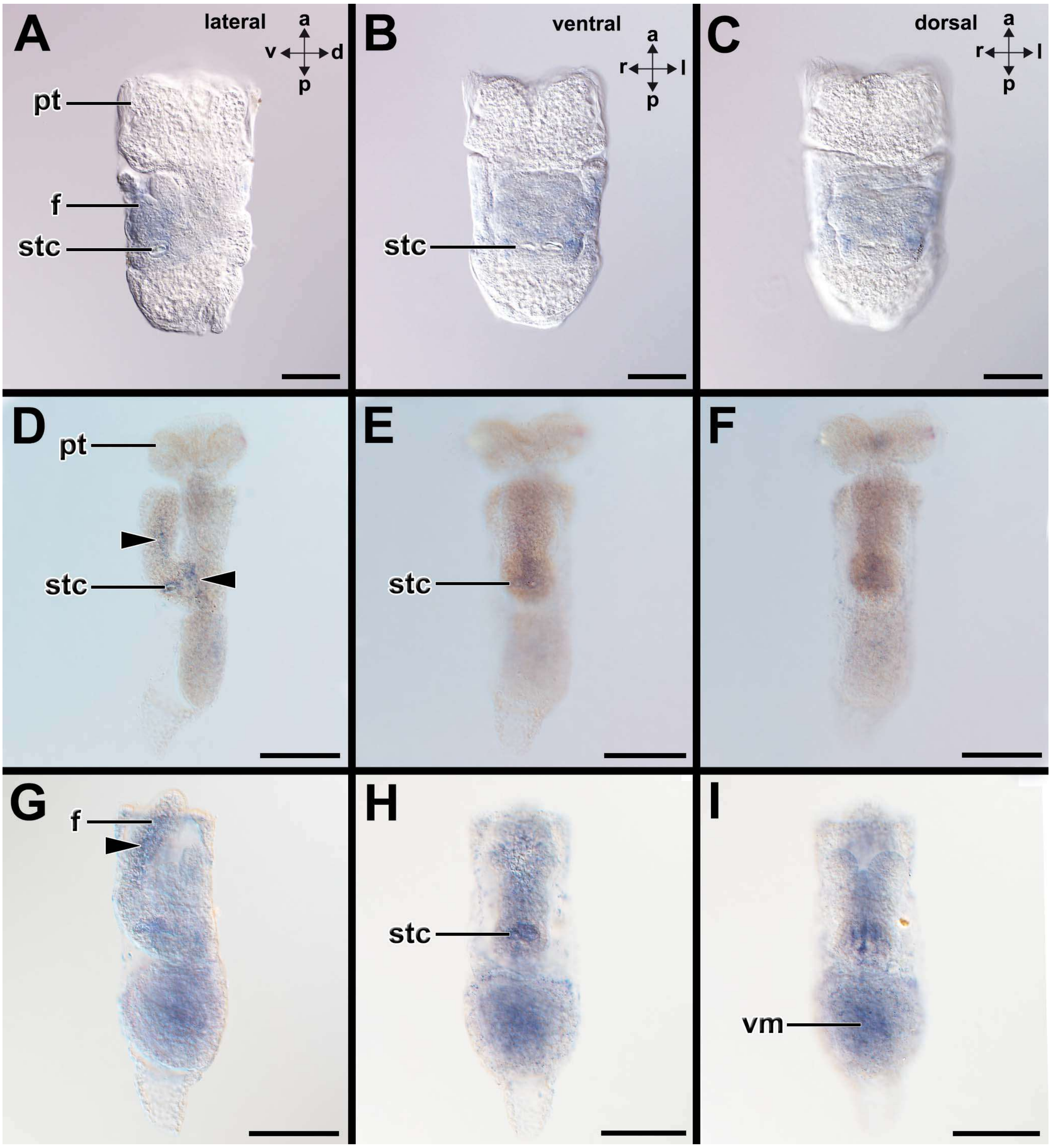

Figure S14. ***Lox5/ Hox6*** **gene expression during late development of the scaphopod *Antalis entalis.*** A-C: Late mid-stage trochophores express *Lox5* lateral, anterior and posterior to the statocysts in the foot. D-F (same orientation as in A-C): Late-stage trochophores express *Lox5* in a domain located in the foot (left arrowhead) and in between the pedal and cerebral ganglia (right arrowhead). G-I (same orientation as in A-C): Postmetamorphic individuals express *Lox5* in the anterior foot (arrowhead). Scale bars: 100 µm.

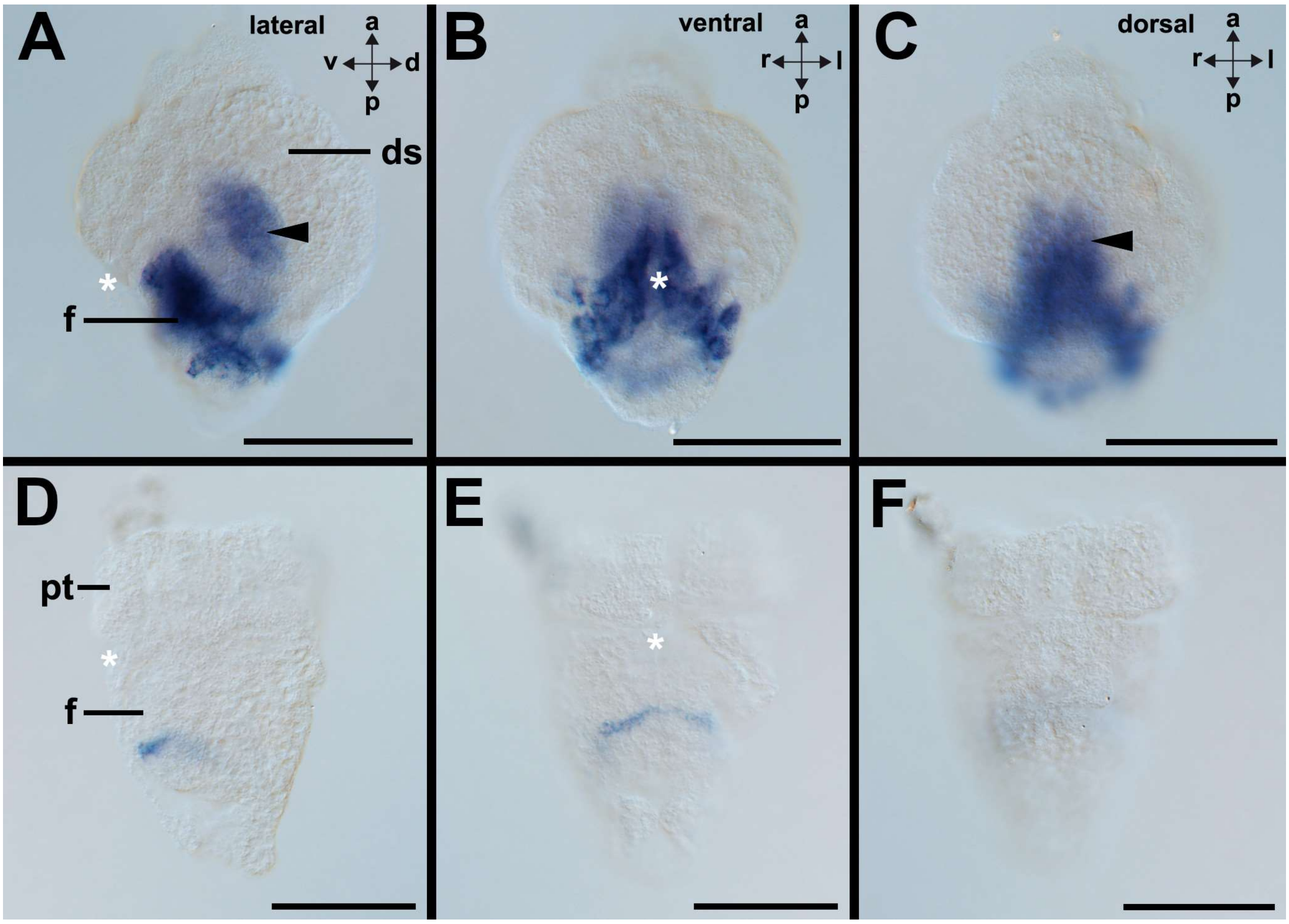

Figure S15. ***Lox4/ Hox8*** **gene expression during early development of the scaphopod** ***Antalis entalis.*** The mouth is labeled with an asterisk. A-C: Early-stage trochophores express *Lox4* in the foot anlage and putative mesoderm in the interior region (arrowheads). D-F (same orientation as in A-C): Early mid-stage trochophores express *Lox4* in cells ventrally to the statocysts and slightly more posterior to the latter. Scale bars: 100 µm.

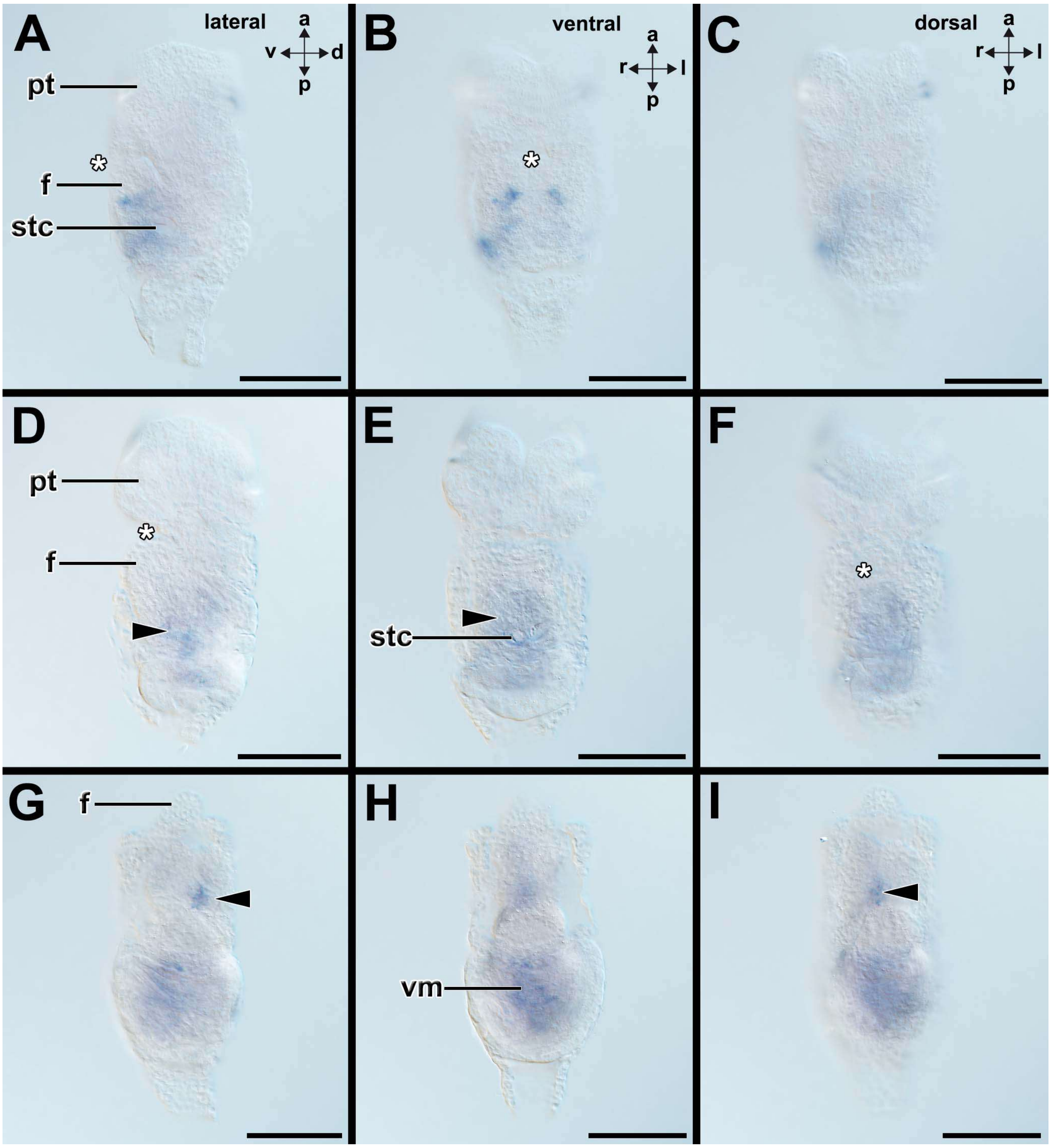

Figure S16. ***Lox4/ Hox8*** **gene expression during late development of the scaphopod** ***Antalis entalis.*** The mouth is labeled with an asterisk. A-C: Late mid-stage trochophores express *Lox4* in two spot-like domains anterior to the statocysts and in two domains posterior to the statocysts in the foot. D-F (same orientation as in A-C): Late-stage trochophores express *Lox4* faintly around the statocysts (arrowheads). G-I (same orientation as in A-C): Postmetamorphic individuals express *Lox4* in the buccal region (arrowheads) and in the visceral mass. Scale bars: 100 µm.

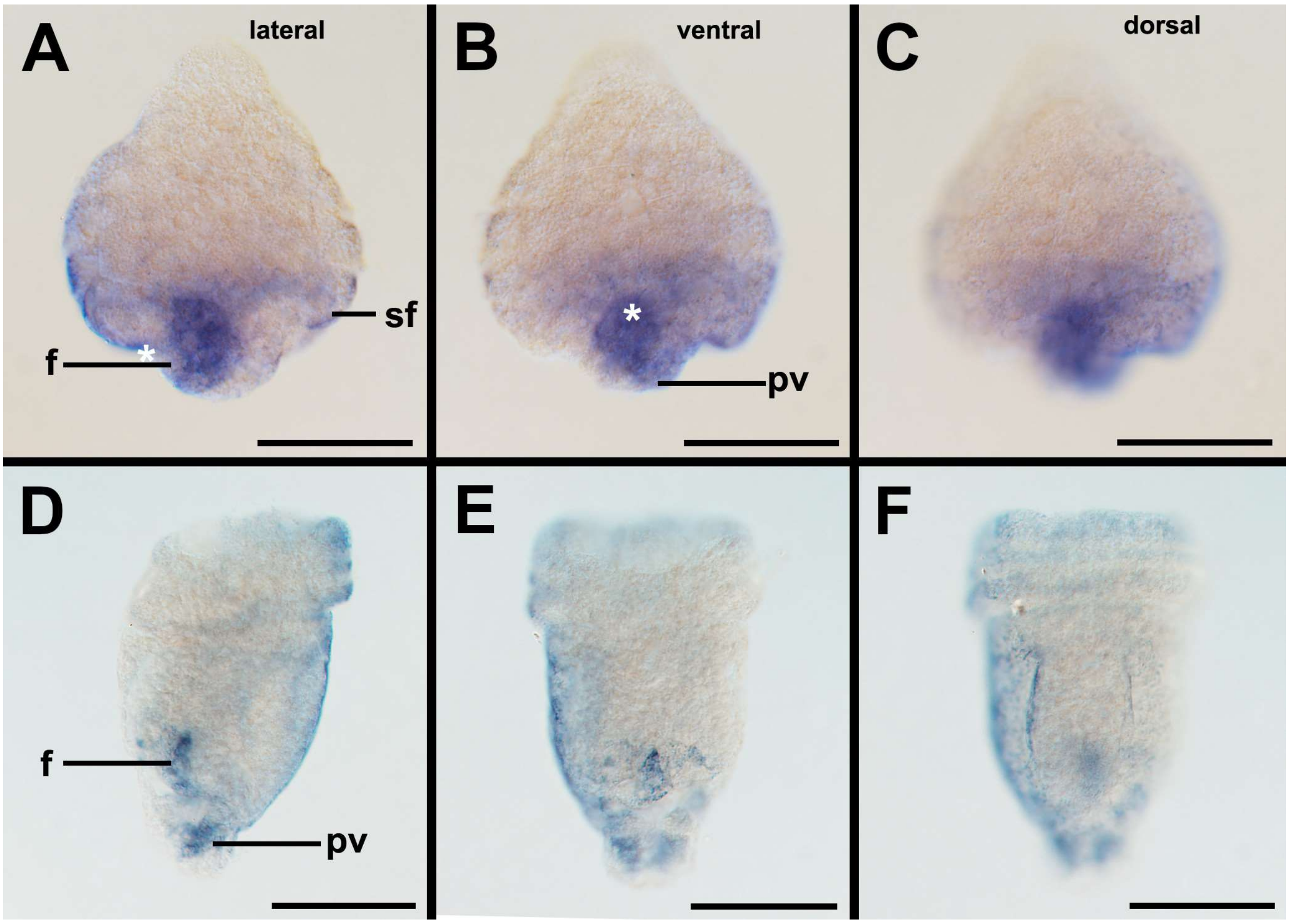

Figure S17. *Post1* gene expression during early development of the scaphopod *Antalis entalis.* The mouth is labeled with an asterisk. A-C: Early-stage trochophores express *Post1* in foot anlage, shell field, and pavilion. D-F (same orientation as in A-C): Early mid-stage trochophores express *Post1* in the posterior foot and the pavilion. Note the weak unspecific staining on the prototroch and mantle. Scale bars: 100 µm.

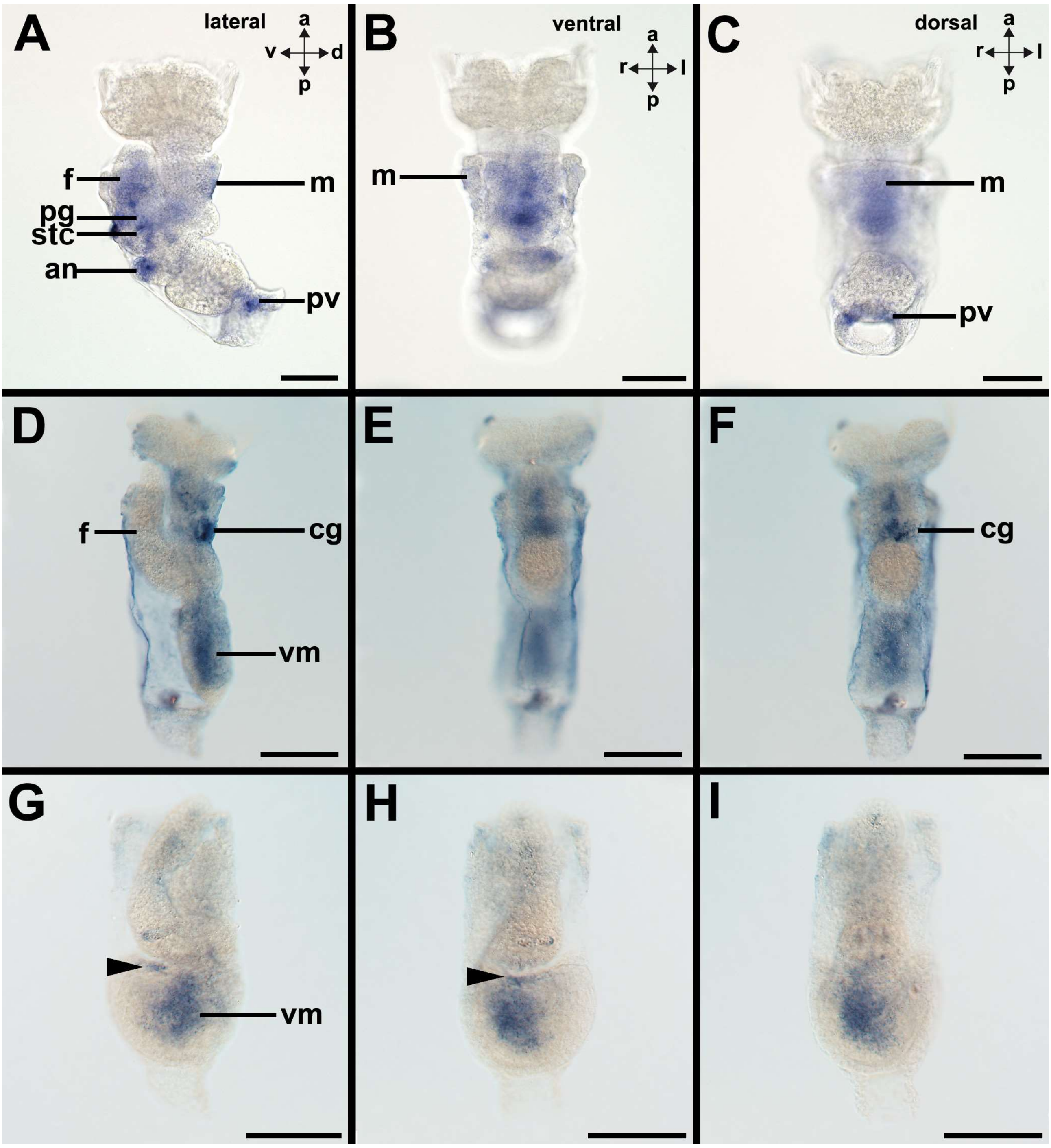

Figure S18. ***Post1*** **gene expression during late development of the scaphopod** ***Antalis entalis.***

A-C: Late mid-stage trochophores express *Post1* in the pavilion, two pedal domains anterior to the statocysts and posterior to the latter and in the anterior lateral and dorsal mantle margin. D-F (same orientation as in A-C): Late-stage trochophores express *Post1* in the cerebral ganglia and the anterolateral mantle margin. G-I (same orientation as in A-C): Postmetamorphic individuals express *Post1* in anterior portion of the intestines (arrowheads) and the visceral mass. Scale bars: 100 µm.

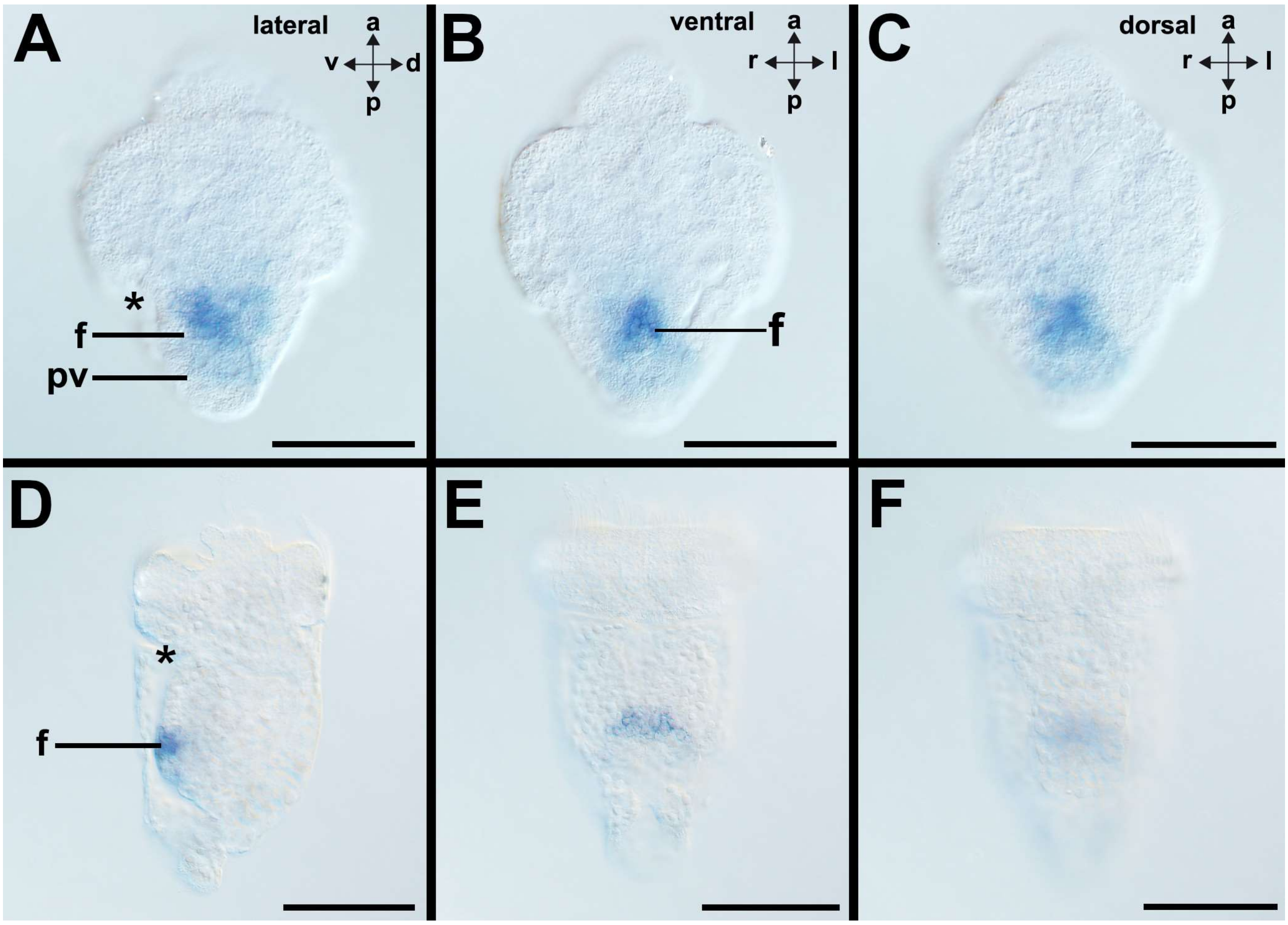

Figure S19. ***Post2*** **gene expression during early development of the scaphopod** ***Antalis entalis.*** The mouth is labeled with an asterisk. A-C: Early-stage trochophores express *Post2* in the anlage of the foot and pavilion. D-F (same orientation as in A-C): Early mid-stage trochophores express *Post2* in a domain extending from anterior to posterior to the statocysts, including the pedal ganglia. Scale bars: 100 µm.

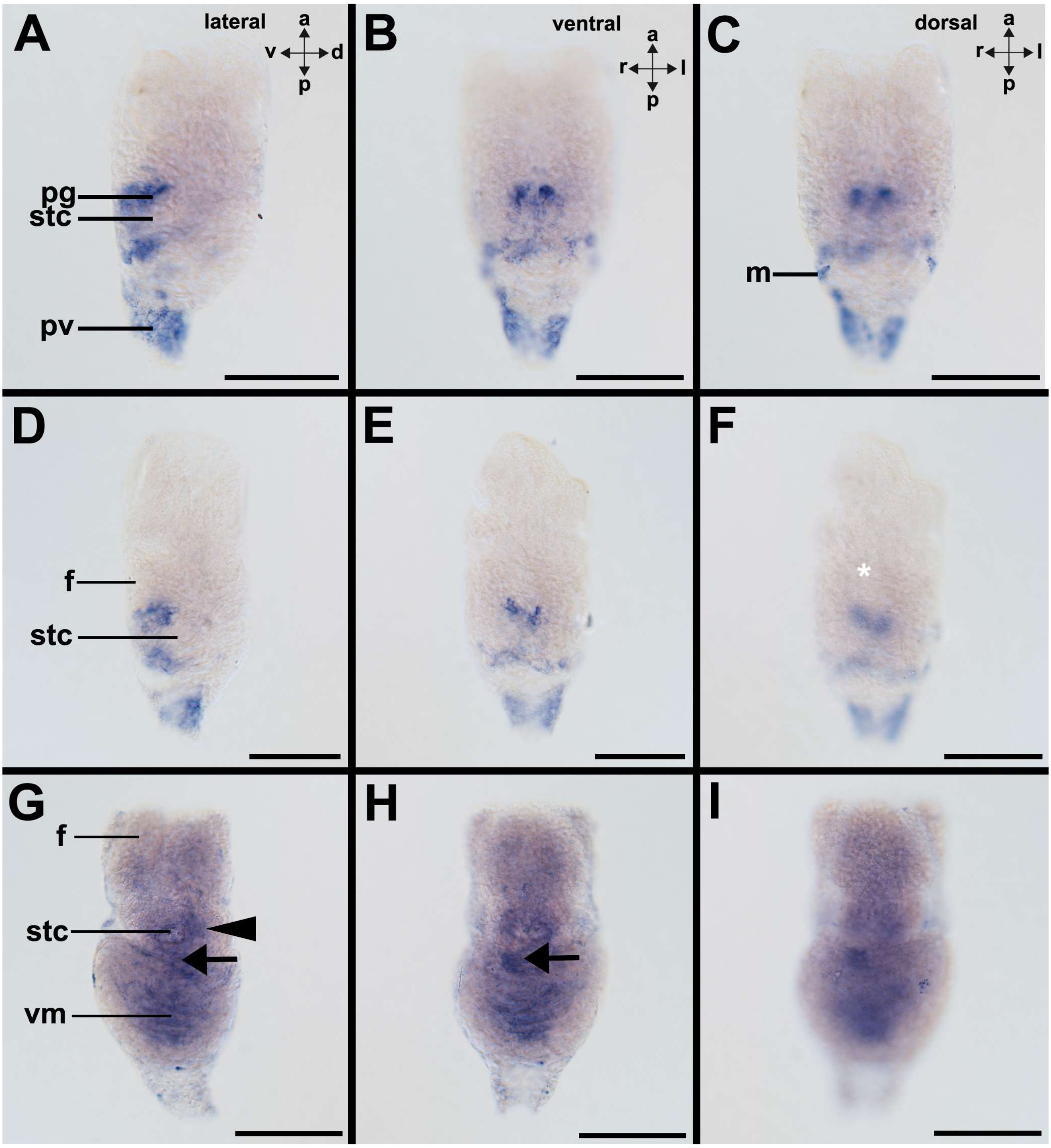

Figure S20. ***Post2*** **gene expression during late development of the scaphopod** ***Antalis entalis.*** The mouth is labeled with an asterisk. A-C: Late mid-stage trochophores express *Post2* in a domain anterior to the statocysts in the foot and posterior to the statocysts with additional expression in the pavilion and mantle (m). D-F (same orientation as in A-C): Late-stage trochophores express *Post2* in domains anterior and posterior to the statocysts. G-I (same orientation as in A-C): Postmetamorphic individuals express *Post2* in the anterior portion of the intestine (arrows) and

dorsally to the statocysts in the posterior foot (arrowheads). Scale bars: 100 µm.

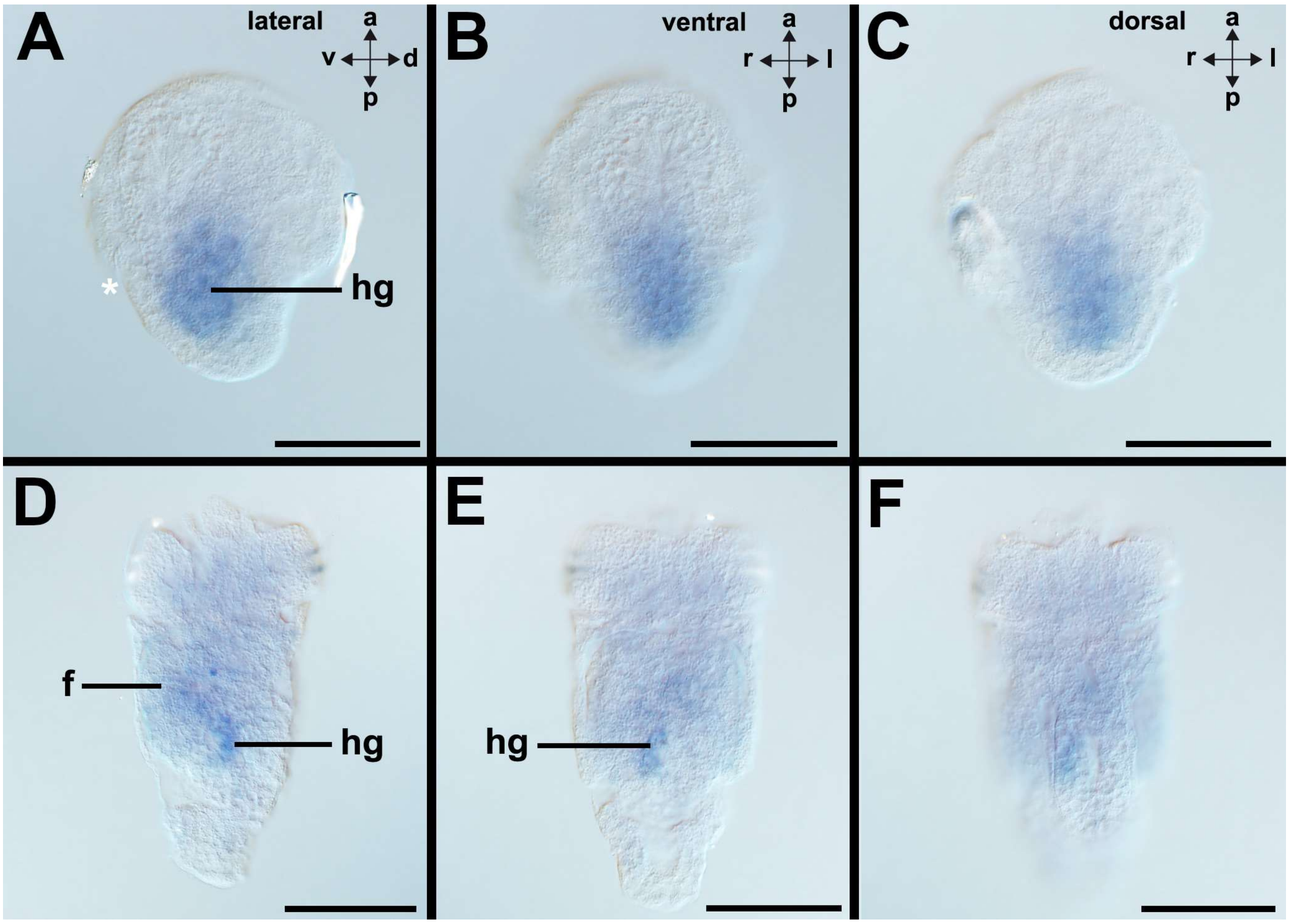

Figure S21. ***Cdx*** **gene expression during early development of the scaphopod** ***Antalis entalis.*** The mouth is labeled with an asterisk. A-C: Early-stage trochophores express *Cdx* in the region of the hindgut. D-F (same orientation as in A-C): Early mid-stage trochophores express *Cdx* in the region of the hindgut. Scale bars: 100 µm.

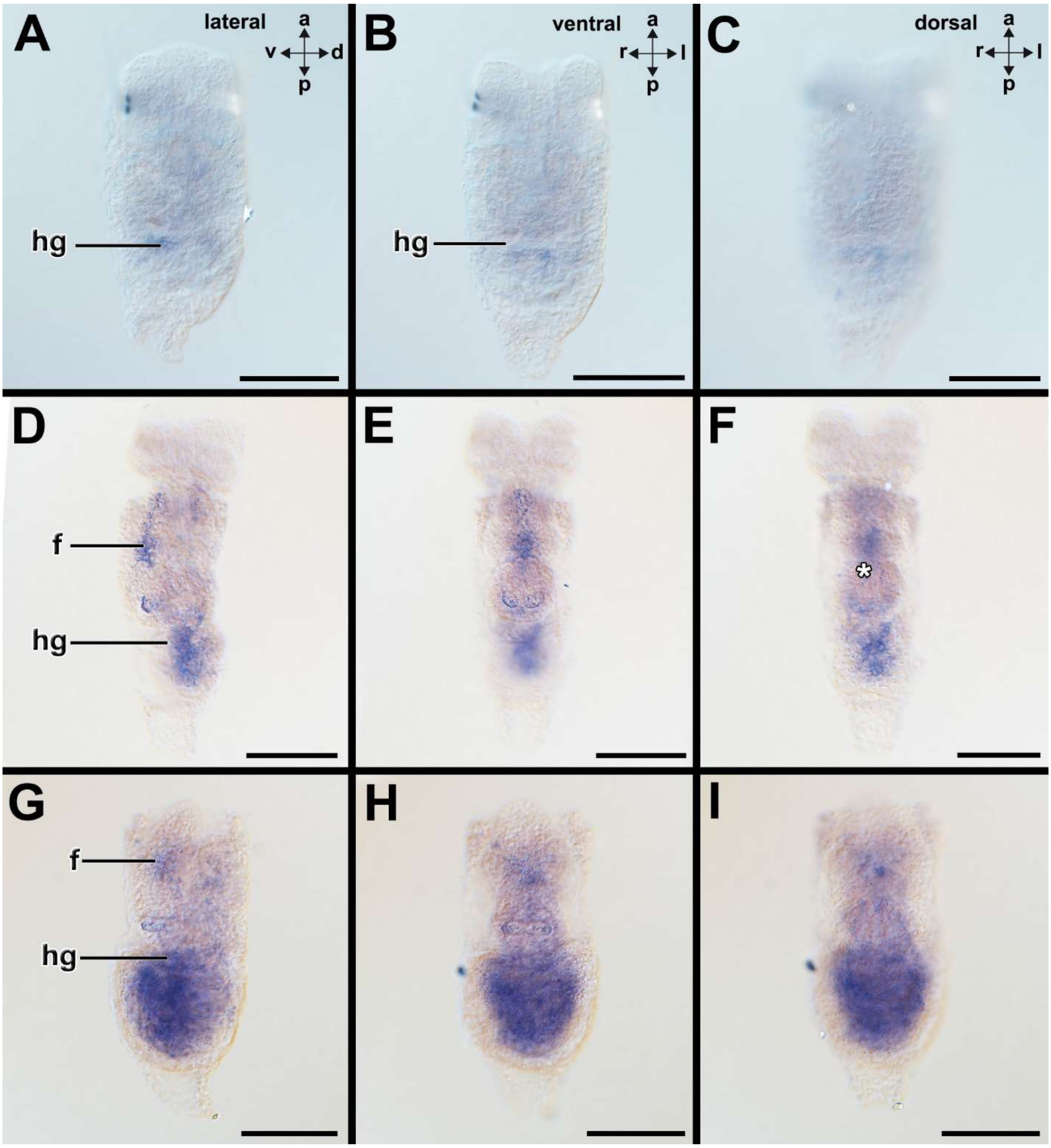

Figure S22. ***Cdx*** **gene expression during late development of the scaphopod** ***Antalis entalis.***

The mouth is labeled with an asterisk. A-C: Late mid-stage trochophores express *Cdx* in the hindgut. D-F (same orientation as in A-C): Late-stage trochophores express *Cdx* in the hindgut and the foot. G-I (same orientation as in A-C): Postmetamorphic individuals express *Cdx* in the foot and the region of the hindgut. Scale bars: 100 µm.

Metazoan elav-like proteins

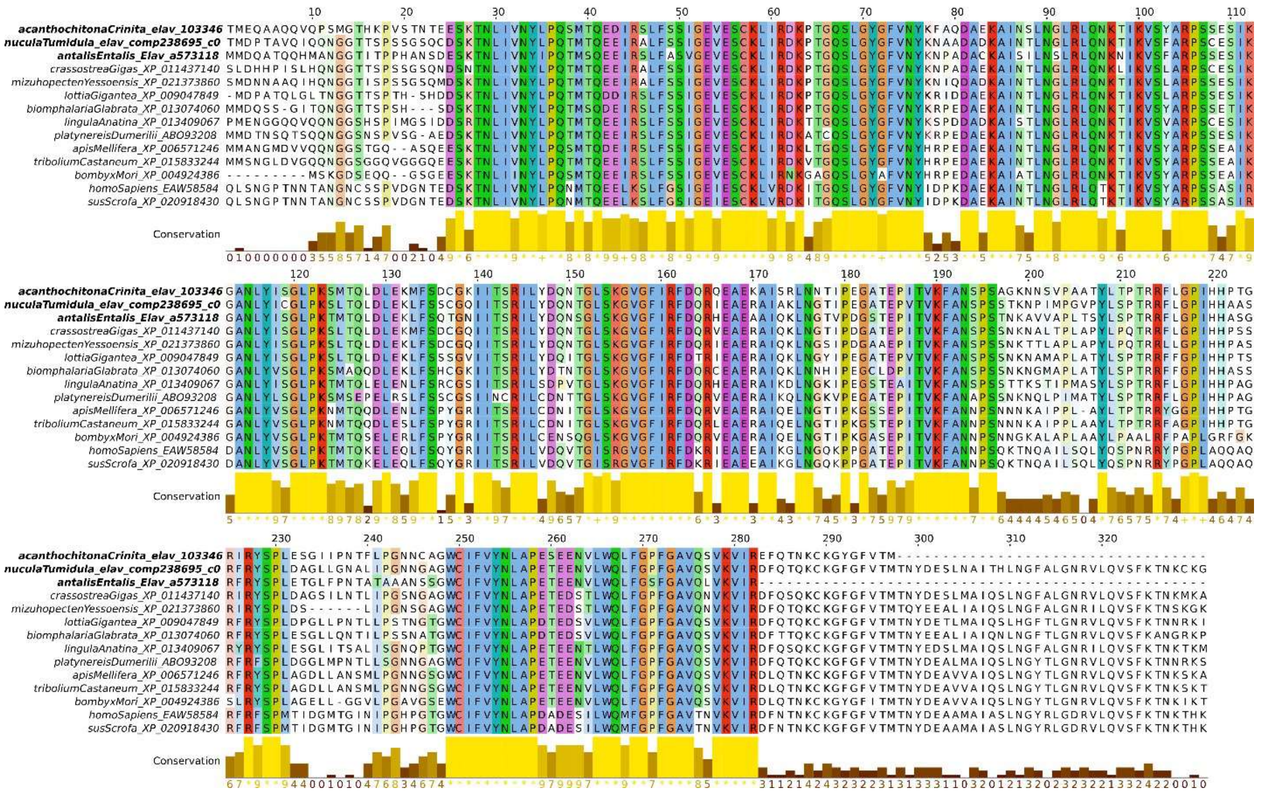

Figure S23. Alignment. Multiple sequence alignment visualisation of the Elav-like proteins using Jalview2 (Waterhouse et al., 2010). Columns are colored following the ClustalX colour scheme based on a minimum percentage identity threshold (>30%). The sequences highlighted in bold are described in this work. The alignment conservation annotation represented by the histograms is the quantitative numerical index reflecting the conservation of the amino acids physico-chemical properties for each column of the alignment.

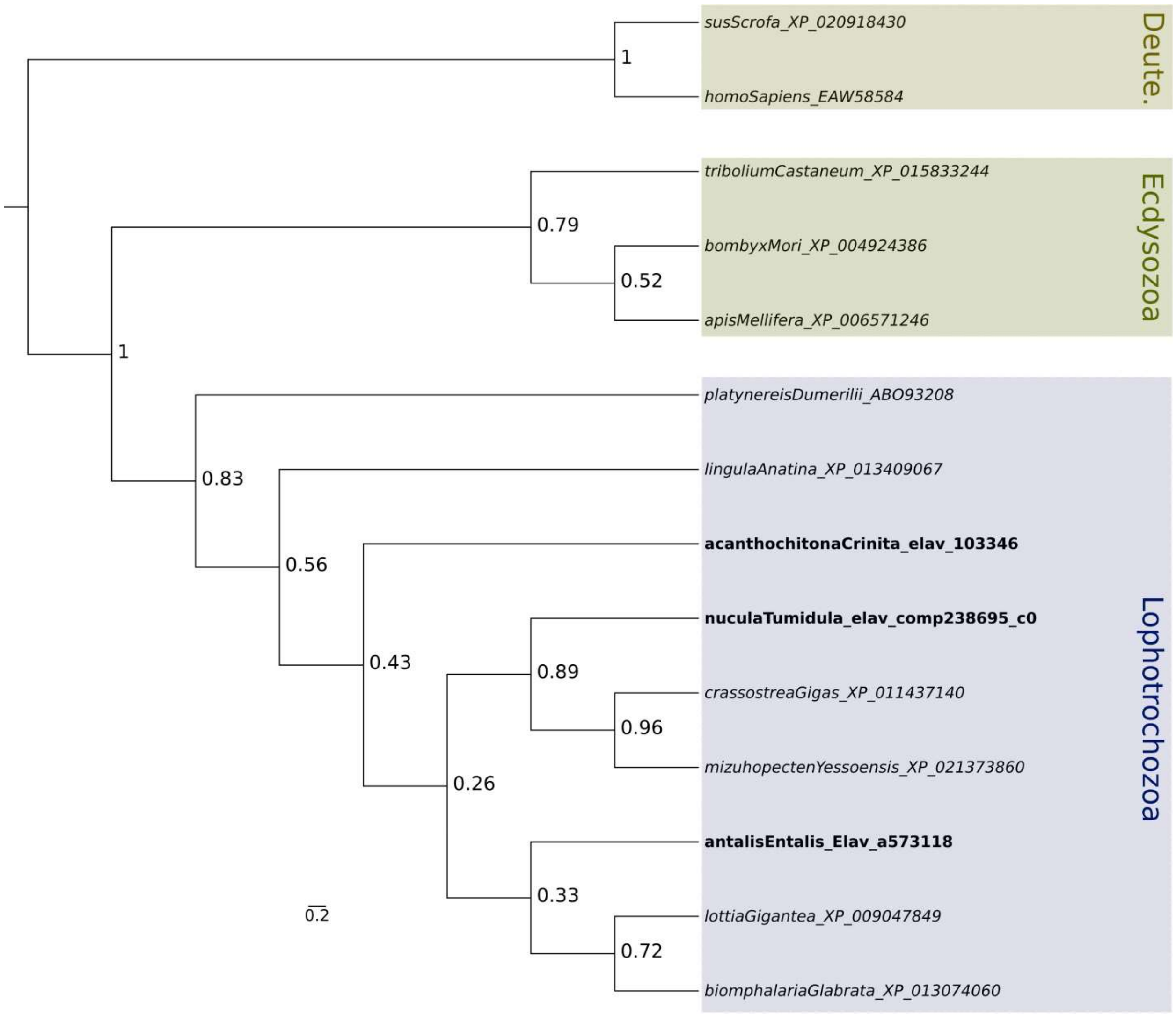

Figure S24. Phylogenetic tree. Maximum likelihood tree of metazoan Elav-like proteins inferred by PhyML software. Support values for the topology (i.e. non-parametric bootstrap with 1000 replicates) are listed as percentages.

Table S1. Hox gene expression domains during molluscan development.

Green: gene expressed; red: gene not expressed as revealed by *in situ* hybridization; ?: no expression pattern available but gene ortholog present; Absent?: no expression pattern/ transcript sequence published; **-**: no transcript found in transcriptomes; *: only gastrula stage investigated. Data based on Lee et al. 2003; Samadi and Steiner 2009, 2010; Fritsch et al. 2015, 2016; Wang et al. 2017; present study.

Molluscan Hox gene expression in the shell field(s) – combined expression of developmental stages

Hox gene/ Clade	Polyplacophora	Scaphopoda	Gastropoda	Bivalvia	Cephalopoda
Hox1				*	
Hox2				*	Absent?
Hox3				*	
Hox4				*	Absent?
Hox5				*	
Lox5				*	
Hox7		-		*	
Lox4				*	
Lox2		?		*	Absent?
Post2				*	
Post1	?			*	Absent?

Molluscan Hox gene expression in the foot and cephalopod arms/ funnel – combined expression of developmental stages

Hox gene/ Clade	Polyplacophora	Scaphopoda	Cephalopoda	Gastropoda	Bivalvia
Hox1					*
Hox2			Absent?		*
Hox3					*
Hox4			Absent?		*
Hox5					*
Lox5					*
Hox7		-			*
Lox4					*
Lox2		?	Absent?		*
Post2					*
Post1	?		Absent?		*

Molluscan Hox gene expression in developing adult nervous system – combined expression of developmental stages

Hox gene/ Clade	Polyplacophora	Scaphopoda	Cephalopoda	Gastropoda	Bivalvia
Hox1					n/a
Hox2			Absent?		n/a
Hox3					n/a
Hox4			Absent?		n/a
Hox5					n/a
Lox5			Probably in arms		n/a
Hox7		-	Probably in arms		n/a
Lox4					n/a
Lox2		?	Absent?		n/a
Post2					n/a
Post1	?		Absent?		n/a

References:

Criscuolo, A., Gribaldo, S. 2010. BMGE (Block Mapping and Gathering with Entropy): a new software for selection of phylogenetic informative regions from multiple sequence alignments. *BMC Evol. Biol.* **13** 210.

Darriba, D., Taboada, G. L., Doallo, R., and Posada, D. 2011 ProtTest 3: fast selection of best-fit models of protein evolution. *Bioinformatics* **27** 1164–1165.

De Oliveira, A. L., Wollesen, T., Kristof, A., Scherholz, M., Redl, E., Todt, C., Bleidorn, C., Wanninger, A. 2016 Comparative transcriptomics enlarges the toolkit of known developmental genes in mollusks. *BMC Genomics* **17** 905.

Fritsch, M., Wollesen, T., de Oliveira, A. L., Wanninger, A. 2015 Unexpected co-linearity of Hox gene expression in an aculiferan mollusk. *BMC Evol. Biol.* **15** 151.

Fritsch, M., Wollesen, T., Wanninger, A. 2016 Hox and ParaHox gene expression in early body plan patterning of polyplacophoran mollusks. *JEZ Part B Mol. Dev. Evol.* **326** 89-104.

Guindon, S., Dufayard, J. F., Lefort, V., Anisimova, M., Hordijk, W., Gascuel, O. 2010 New algorithms and methods to estimate maximum-likelihood phylogenies: assessing the performance of PhyML 3.0. *Syst. Biol.* **59** 307-21.

Jékely, G. & Arendt, D. 2007 Confocal detection of NBT/BCIP *in situ* hybridization samples by reflection microscopy. *Biochemica* **4** 12-14.

Katoh, K. and Standley, D. M. 2014 MAFFT: iterative refinement and additional methods. *Meth. Mol. Biol.* **1079** 131–146.

Lee, P. N., Callaerts, P., de Couet, H. G., Martindale, M. Q. 2003 Cephalopod *Hox* genes and the origin of morphological novelties. *Nature* **424**, 1061-1065.

Rambaut, A. 2008 FigTree v1.4.1. http://tree.bio.ed.ac.uk/figtree.

Samadi, L. & Steiner, G. 2009 Involvement of *Hox* genes in shell morphogenesis in the

encapsulated development of a top shell gastropod (*Gibbula varia* L.). *Dev. Genes Evol.* **219** 523–530.

Samadi, L. & Steiner, G. 2010 Expression of *Hox* genes during the larval development of the snail, *Gibbula varia* (L.)-further evidence of non-colinearity in molluscs. *Dev. Genes Evol.* **220** 161-172.

Wang, S., *et al.* 2017 Scallop genome provides insights into evolution of bilaterian karyotype and development. *Nature Ecol. Evol.* **1** 0120.

Waterhouse, A. M., Procter, J. B., Martin, D. M. A., Clamp, M., and Barton, G. 2009 Jalview Version 2 - a multiple sequence alignment editor and analysis workbench. *Bioinformatics* **25** 1189-1191.

Wollesen, T., Rodríguez Monje, S. V., Todt, C., Degnan, B. M. & Wanninger, A. 2015 Ancestral role of *Pax2/5/8* in molluscan brain and multimodal sensory system development. *BMC Evol. Biol.* **15** 231.
